# Inhibition of ferroptosis in inflammatory macrophages alleviates intestinal injury in neonatal necrotizing enterocolitis

**DOI:** 10.1038/s41420-025-02665-9

**Published:** 2025-08-05

**Authors:** Leiting Shen, Jiayu Chen, Jinfa Tou

**Affiliations:** Department of Neonatal Surgery, Children’s Hospital, Zhejiang University School of Medicine, National Clinical Research Center for Child Health, Hangzhou, China

**Keywords:** Paediatric research, Inflammatory diseases, Immune cell death

## Abstract

Neonatal necrotizing enterocolitis (NEC) is a severe gut disease primarily affecting preterm infants, driven significantly by inflammatory macrophages. This study combined bioinformatics (single-cell/tissue RNA sequencing) and experiments to identify key macrophage changes in NEC. Analysis revealed substantial macrophage infiltration in NEC tissues. These macrophages were highly inflammatory and strongly linked to cell death pathways (ferroptosis, pyroptosis, apoptosis), with scores significantly higher than controls and correlating with inflammation. In vitro, LPS-stimulated inflammatory macrophages showed elevated ferroptosis, evidenced by cell rupture, death, increased ACSL4, decreased GPX4, iron overload, lipid peroxidation, and heightened cytokine release. Critically, the ferroptosis inhibitor Ferrostatin-1 (Fer-1) reversed these effects. While LPS alone didn’t kill intestinal epithelial cells, supernatant from LPS-stimulated macrophages significantly increased intestinal epithelial cell death. Fer-1 inhibition of macrophage ferroptosis prevented this epithelial damage. In vivo, a mouse NEC model (induced by hypersomolar feeding, hypoxia, cold) displayed macrophage infiltration, inflammation, and elevated ferroptosis markers. Intraperitoneal Fer-1 administration improved intestinal injury in NEC mice. This study demonstrates that macrophage ferroptosis is a critical driver of NEC inflammation and tissue damage. Inhibiting ferroptosis with Fer-1 effectively reduces both macrophage death and subsequent intestinal epithelial injury, mitigating NEC progression. These findings highlight macrophage ferroptosis as a key therapeutic target for NEC, offering a foundation for new treatment strategies.

## Introduction

Neonatal necrotizing enterocolitis (NEC) is a severe gastrointestinal complication with a high incidence, mortality rate, and economic burden [1, 2]. NEC manifests in 1–7% of preterm neonates, with the highest incidence observed at 29–32 weeks post-conceptional age [3]. Notably, while general survival rates and developmental prognoses in preterm populations have shown progressive improvement, NEC-associated mortality rates in the Neonatal Intensive Care Unit (NICU) have demonstrated persistent stability, currently ranging from 20 to 30% [4]. Alarmingly, survivors frequently develop immediate and long-term comorbidities, most notably intestinal failure manifested as short bowel syndrome (SBS), infective shock, persistent growth retardation, and compromised neurocognitive development [5]. The lack of targeted therapies for NEC has hindered breakthroughs in treatment research. Therefore, it is imperative to investigate the mechanisms underlying NEC pathogenesis to identify key regulatory targets.

The etiology of NEC remains unclear, with current evidence suggesting associations with intestinal immaturity, dysfunctional intestinal activity, and exposure to hyperosmolar feeds/formula [6]. Pathologically, NEC presents as the destruction of the intestinal epithelial barrier accompanied by epithelial cell damage and death [7]. Molecularly, pathogenic microorganisms breach the immature intestinal barrier via pattern recognition receptors (PRRs) detecting pathogen-associated molecular patterns (PAMPs) or danger-associated molecular patterns (DAMPs), triggering abnormal activation of innate immunity [8]. As the primary component of innate immunity, intestinal macrophages exhibit remarkable functional plasticity in early gut development, pathogen resistance, barrier maintenance, and microbiota regulation [9]. Under different stimuli, these macrophages can differentiate into distinct subtypes with varying biological functions. Preterm infants are particularly susceptible to high LPS exposure, which provides sufficient stimulation to the immature intestinal mucosa and facilitates M1 macrophage polarization [9, 10]. Upon LPS stimulation, macrophages activate TLR4/NF-κB signaling pathways and release proinflammatory cytokines (IL-1β, IL-6, TNF-α) that create an inflammatory microenvironment, exacerbating intestinal injury [10]. Our previous studies demonstrated activation of the NLRP3/caspase-1/IL-1β pathway in NEC-associated macrophages [11, 12]. Notably, while LPS alone caused minimal damage to intestinal epithelial cells (IECs), pyroptotic macrophages induced significant IEC death via inflammatory mediators [11]. These findings suggest that modulating macrophage inflammatory activation and cytokine release may alleviate NEC progression. However, the crosstalk mechanisms between macrophages and intestinal epithelium remain incompletely understood. Our prior work focused specifically on macrophage pyroptosis, leaving other potential mechanisms of inflammatory macrophages in NEC requiring further investigation.

With the rapid advancement of omics technologies, conventional bulk RNA sequencing (bulk RNA-seq) has emerged as a pivotal omics approach for investigating critical molecular targets. This technique has been increasingly employed in disease research to identify significant gene expression changes during pathogenesis and to pinpoint associated molecular targets. While several transcriptomic studies using NEC intestinal tissue samples have revealed potential mechanisms and candidate genes [13, 14], bulk RNA-seq primarily assesses the averaged gene expression across heterogeneous cell populations, limiting its capacity to delineate cell type-specific alterations. Single-cell RNA sequencing (scRNA-seq) effectively overcomes this limitation by enabling precise characterization of critical cell subtypes and their differential gene expression profiles. Leveraging this technological advantage, the present study analyzed scRNA-seq data from NEC patients, building upon prior research foundations to specifically investigate macrophage-associated dynamics during NEC progression. Additionally, we conducted wet experiments encompassing clinical samples, mouse models, and cell models to achieve both exploratory investigations and experimental validation. This study will help to further clarify the key targets and mechanism changes of macrophages based on previous studies, and provide a theoretical basis for targeted therapy of NEC.

## Results

### Macrophage accumulates in the intestine of NEC patients and mice

**Fig. 1:**
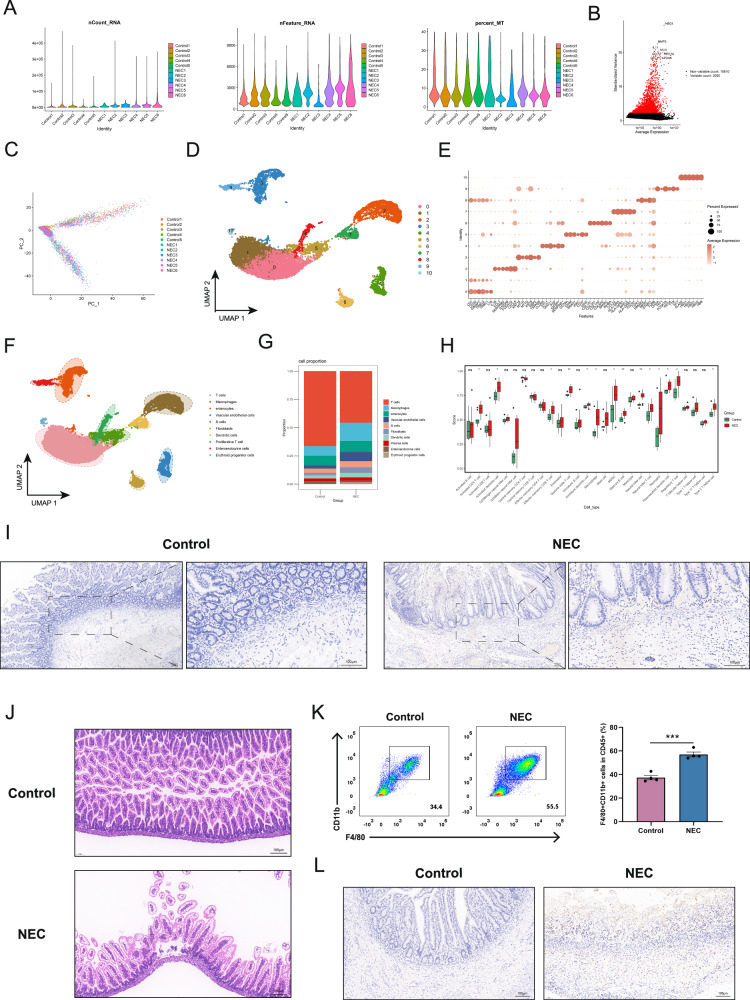
Macrophage accumulates in intestines of NEC patients and mice. **A** 21,833 cells and 14,298 genes were obtained after scRNA-seq QC. **B** Variance plots showing changes in gene expression in cells. **C** PCA analysis revealed a uniform distribution of the samples. **D**–**F** UMAP dimensionality reduction identified 11 distinct cell populations. By combining the top marker genes for each cluster and referencing the original literature, 10 different cell types were annotated. **G** Cell proportion stack plot. **H** Immune cell infiltration scores of bulk RNA-seq data from the control and NEC groups were evaluated using ssGSEA. **I** Immunohistochemical staining results of CD68 (macrophage marker) in intestinal tissues from control and NEC group patients (scale bar: 100 μm). **J** Representative HE staining images of intestinal tissues from control and NEC group mice (scale bar: 100 μm). **K** Flow cytometry results showing the percentage of macrophages among CD45+ cells in the intestinal tissues of control and NEC group mice. The bar graph illustrates the intestinal macrophages in CD45+ cells in the two groups of mouse intestinal tissues. **L** Representative immunohistochemical staining images of CD68 (macrophage marker) in the intestinal tissues of control and NEC group mice. Data are mean ± SEM from 4 (**K**). ***p < 0.001.

First, we performed quality control based on the criteria of RNA nCount > 1000, RNA nFeature > 300, and percentage of mitochondrial genes < 40. This resulted in 21,833 cells and 14,298 genes for further analysis (Fig. 1A). Using the “FindVariableFeatures” function, we identified 2000 highly variable genes (Fig. 1B). PCA analysis revealed a uniform distribution of the single-cell data with no significant batch effects (Fig. 1C). UMAP clustering of the data identified 11 distinct cell populations (Fig. 1D). After annotating the top marker genes, we classified the cells into 10 groups: T cells, macrophages, enterocytes, vascular endothelial cells, B cells, fibroblasts, dendritic cells, proliferative T cells, enteroendocrine cells, and erythroid progenitor cells (Fig. 1E, F). Building on previous studies, we focused on changes in macrophages. The cell proportion stack plot revealed that the overall proportion of macrophages in the NEC group was higher than that in the control group (Fig. 1G). Similar results were obtained in the scRNA-seq dataset GSE178088 (Fig. S1A–C). Subsequently, immune infiltration analysis in bulk RNA-seq data (merged GSE46619 and GSE64801 datasets) showed that the macrophage infiltration score in the NEC group was significantly higher than that in the control group (Fig. 1H). Further validation was carried out in clinical samples and mouse models. Immunohistochemical analysis of clinical samples demonstrated that, compared to the control group, CD68+ macrophages were extensively infiltrated in the mucosa and submucosa in the NEC group (Fig. 1I). Additionally, we established the NEC mouse model using hypoxia, cold stimulation, and hyperosmotic formula feeding. H&E staining revealed significant villus shedding, disruption of intestinal structure, and partial separation in the NEC group compared to the control group (Fig. 1J). Flow cytometry showed that, compared to the control group, the proportion of F4/80+CD11b+ macrophages among the CD45+ cells in the intestinal tract was significantly higher in the NEC group (Fig. 1K and S1D). Furthermore, immunohistochemical staining of mouse intestines showed a significant increase in the expression of the macrophage marker CD68 in the NEC group compared to the control group (Fig. 1L). The above results suggest significant macrophage infiltration in the intestinal tissue of NEC.

### The major macrophage population in NEC exhibits high proinflammatory activity

**Fig. 2:**
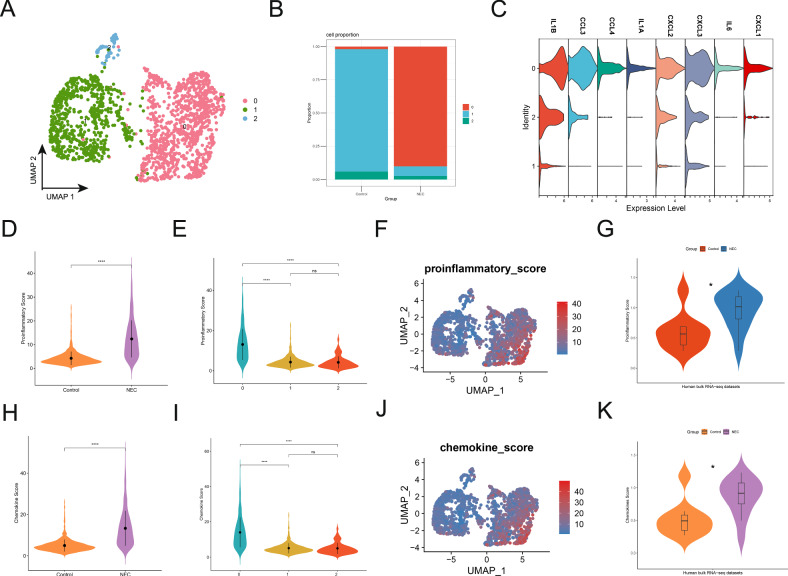
The major macrophage population in NEC exhibits high proinflammatory activity. **A** Reclustering of macrophages identified three distinct macrophage subtypes. **B** Stacked bar plot depicting the proportions of macrophage subtypes. **C** The violin plot illustrates the expression of several proinflammatory factors and chemokines across different macrophage subpopulations. **D** The violin plot illustrates the proinflammatory scores of macrophages between the control and NEC groups. **E** The violin plot shows the proinflammatory scores of different macrophage subpopulations. **F** The UMAP plot shows the distribution of proinflammatory scores from low to high. **G** Proinflammatory scores in the control and NEC groups from bulk RNA-seq. **H** The violin plot shows the chemotactic scores of macrophages between the control and NEC groups. **I** The violin plot shows the chemokine scores of different macrophage subpopulations. **J** The UMAP plot shows the distribution of chemokine scores from low to high. **K** Chemokine scores in the control and NEC groups from bulk RNA-seq.

Through the research in the first section, we observed significant macrophage infiltration in the intestines of NEC. To further investigate the changes in macrophages associated with NEC, we extracted macrophages from scRNA-seq data and performed subclustering, which identified three distinct macrophage subpopulations (Fig. 2A). The cell proportion stacked bar plot revealed that in the NEC group, macrophages were predominantly in subgroup 0, whereas in the control group, macrophages were mainly in subgroup 1 (Fig. 2B). In the scRNA-seq validation dataset GSE178088, we reclustered the macrophages and identified four subgroups, with subgroup 0 representing the predominant macrophage population in NEC and subgroup 1 corresponding to the control group (Fig. S2A, B). To further define the nature of the predominant macrophage population (subgroup 0) in the NEC group, we examined key inflammatory factors and chemokines, such as IL-1β, IL-6, CCL3, CCL4, etc. The expression levels of these genes were significantly higher in the predominant macrophage population (subgroup 0) of the NEC group compared to the control group’s predominant macrophage population (subgroup 1) (Fig. 2C). Similar findings were obtained from the validation dataset GSE178088 (Fig. S2C). Additionally, we performed gene set scoring for proinflammatory and chemokine gene sets in the scRNA-seq. The results revealed that the proinflammatory scores in the NEC group were significantly higher than in the control group, with subgroup 0 macrophages in the NEC group exhibiting markedly elevated proinflammatory scores compared to subgroup 1 macrophages in the control group (Fig. 2D–F). Furthermore, ssGSEA analysis of bulk RNA-seq data showed consistent results with the single-cell data (Fig. 2G). Chemokine gene set scoring analysis in both single-cell and bulk RNA-seq data also revealed significantly higher chemokine scores in the NEC group, with subgroup 0 macrophages in the NEC group displaying substantially higher chemokine scores than subgroup 1 macrophages in the control group (Fig. 2H–K). Consistent conclusions were obtained from the gene set scoring analysis of proinflammatory and chemokine gene sets in the scRNA-seq validation dataset GSE178088 (Fig. S2D–I). These findings indicate that compared to the control group, the predominant macrophage phenotype in NEC has shifted, with macrophages in the NEC group exhibiting enhanced proinflammatory and chemotactic properties.

### The predominant macrophage population in NEC is identified as highly apoptotic, pyroptotic, and ferroptotic macrophages

**Fig. 3:**
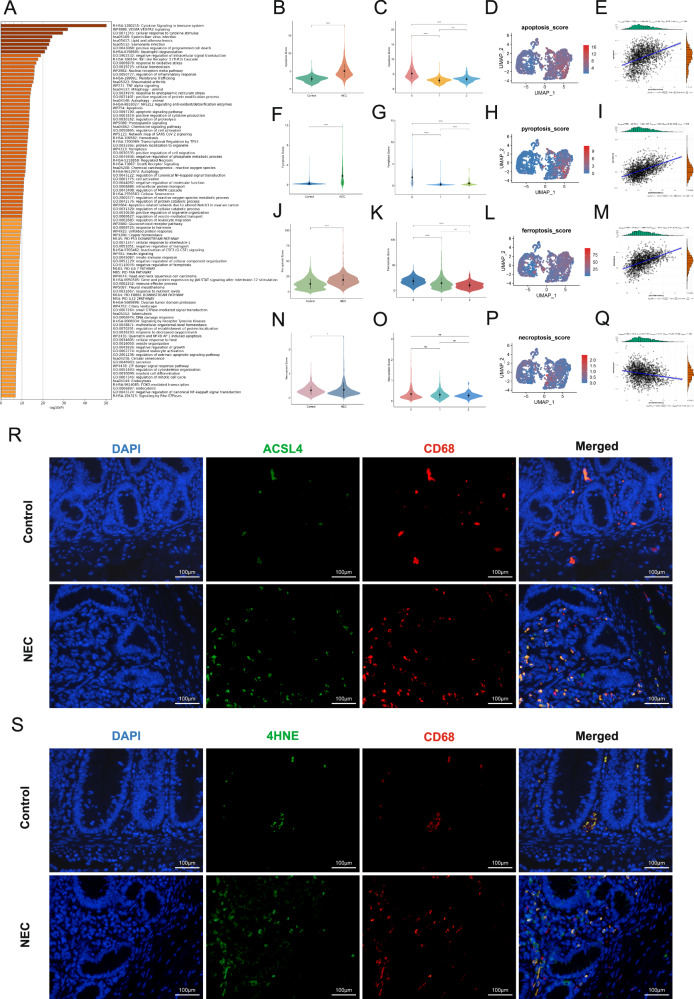
The predominant macrophage population in NEC is identified as highly apoptotic, pyroptotic, and ferroptotic macrophages. **A** Enrichment analysis of top marker genes for the main macrophage population in the NEC group. **B**, **F**, **J**, **N** Violin plot of macrophage apoptosis, pyroptosis, ferroptosis, and necroptosis scores between the NEC and control groups. **C**, **G**, **K**, **O** Violin plot of apoptosis, pyroptosis, ferroptosis, necroptosis scores for different macrophage subpopulations. **D**, **H**, **L**, **P** UMAP plot of apoptosis, pyroptosis, ferroptosis, and necroptosis scores for different macrophage subpopulations. **E**, **I**, **M**, **Q** Scatter plot of the correlation between apoptosis, pyroptosis, ferroptosis, necroptosis scores and proinflammatory scores. **R** Immunofluorescence staining shows the co-localization of ACSL4 and CD68-positive cells in intestinal tissue samples from NEC and control patients (scale bar: 100 µm). **S** Immunofluorescence staining shows the co-localization of 4HNE and CD68-positive cells in intestinal tissue samples from NEC and control patients (scale bar: 100 µm).

To investigate the major changes in macrophages during NEC, we extracted the top marker genes of subgroup 0 macrophages and performed enrichment analysis. The enrichment results suggested that these key altered genes were associated with cell death, such as positive regulation of programmed cell death, apoptosis, ferroptosis, and death receptor signaling (Fig. 3A). Based on these findings and previous literature, we focused on four classic forms of cell death—apoptosis, necroptosis, pyroptosis, and ferroptosis—to further clarify the changes in macrophage death in NEC. After retrieving the relevant genes from the GeneCards and KEGG databases, we performed gene set scoring on the single-cell dataset. The results showed that the apoptosis score of macrophages in the NEC group was significantly higher than that of the control group (Fig. 3B). Furthermore, the apoptosis score of subgroup 0 macrophages in the NEC group was significantly higher than that of subgroup 1 macrophages in the control group (Fig. 3C, D). Additionally, correlation analysis revealed a significant positive correlation between the apoptosis score and the proinflammatory score (Fig. 3E). The gene set scores for pyroptosis and ferroptosis also yielded consistent results with apoptosis (Fig. 3F–M). However, although the necroptosis score showed a slight difference between the NEC and control groups, there was no significant difference between the macrophage subgroups in the NEC group and those in the control group (Fig. 3N–Q). Interestingly, consistent conclusions were also found in the single-cell validation dataset, GSE178088, which confirms the reliability of the conclusion (Fig. S3A-P). These results highlight the importance of pyroptosis, apoptosis, and ferroptosis in macrophages during the progression of NEC. We have previously focused on the key role of pyroptosis in macrophages during NEC and have conducted detailed validation, which will not be elaborated on further here [11, 12]. Ferroptosis is an iron-dependent form of programmed cell death characterized by the accumulation of lipid peroxides, loss of membrane integrity, and inflammatory responses. Lipids containing polyunsaturated fatty acids (PUFAs) are particularly susceptible to peroxidation during ferroptosis. ACSL4, a key enzyme in lipid metabolism, serves as a critical regulator of ferroptosis [15–18]. ACSL4 facilitates the incorporation of PUFAs (such as arachidonic acid) into phospholipids, a process that amplifies lipid peroxidation. Elevated expression of ACSL4 renders cells more susceptible to ferroptosis. Immunofluorescence results showed increased ACSL4-positive (green) and CD68-positive (red) cells in the intestinal tissues of NEC patients compared to controls (Fig. 3R). 4-Hydroxynonenal (4-HNE) is a primary product of lipid peroxidation in ferroptosis. Immunofluorescence staining revealed increased co-localization of CD68 and 4-HNE in the intestinal tissue of the NEC group compared with the control group (Fig. 3S). These findings suggest the occurrence of macrophage ferroptosis in NEC. Apoptosis is a key form of programmed cell death, with caspase-3 serving as the main executor. When cleaved into its active form (cleaved caspase3), it directly participates in the cell death process. Immunofluorescence results showed an increase in cleaved caspase3-positive (green) and CD68-positive (red) cells in the intestinal tissues of NEC patients compared to the Control group (Fig. S4). The immunofluorescence results were consistent with the previous analysis.

### Pseudotime analysis reveals increased macrophage apoptosis and ferroptosis during NEC progression

**Fig. 4:**
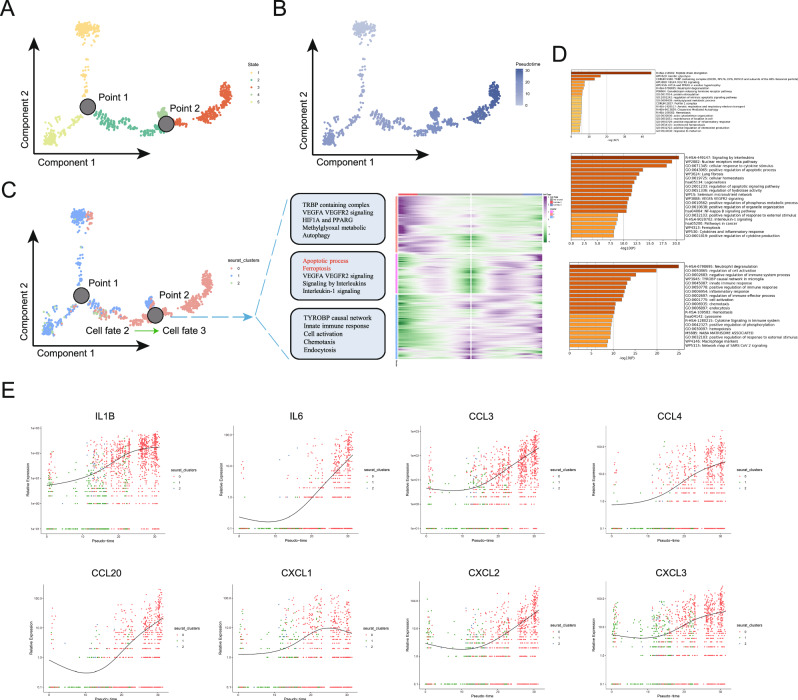
Pseudotime analysis reveals increased macrophage apoptosis and ferroptosis during NEC progression. **A** Pseudotime analysis divided macrophages into five distinct states. **B** The pseudotime trajectory of macrophages. **C** The differentiation trajectories of different macrophage subpopulations and BEAM analysis at node 2. **D** Enrichment analysis of the three clusters of differentially expressed genes at node 2. **E** The gene expression changes of several proinflammatory factors and chemotactic factors from the beginning to the end of the pseudotime process.

To explore the temporal changes in macrophages, we performed pseudotime analysis. This analysis identified two key nodes, categorizing macrophages into five distinct states (Fig. 4A). During the pseudotime progression, the non-inflammatory, low-death macrophage population 1 in the control group gradually transitioned into the highly inflammatory, high-death macrophage population 0 in the NEC group (Fig. 4B, C). Our primary focus was on the NEC macrophage population 0, so we further examined the cellular fate changes at node 2 (where the cell fate changes from fate 2 to fate 3, fully transitioning into NEC macrophage population 0). We conducted BEAM analysis at node 2, identified the corresponding differentially expressed genes, and clustered them into three gene categories, each playing distinct biological roles. Enrichment analysis of these three gene clusters revealed that the first cluster was associated with signaling pathways such as HIF1A, PPARG, and autophagy, the second cluster was linked to programmed cell death pathways like apoptosis and ferroptosis, and the third cluster was involved in functions such as cell activation, chemotaxis, and phagocytosis (Fig. 4D). Additionally, we selected several key inflammatory and chemotactic factors to analyze their gene expression changes throughout the pseudotime process, from start to finish. The results indicated that, as pseudotime progressed from the normal state to the NEC state, the expression of these proinflammatory factors gradually increased (Fig. 4E).

### Inhibiting ferroptosis in inflammatory macrophages effectively alleviates intestinal epithelial cell death

**Fig. 5:**
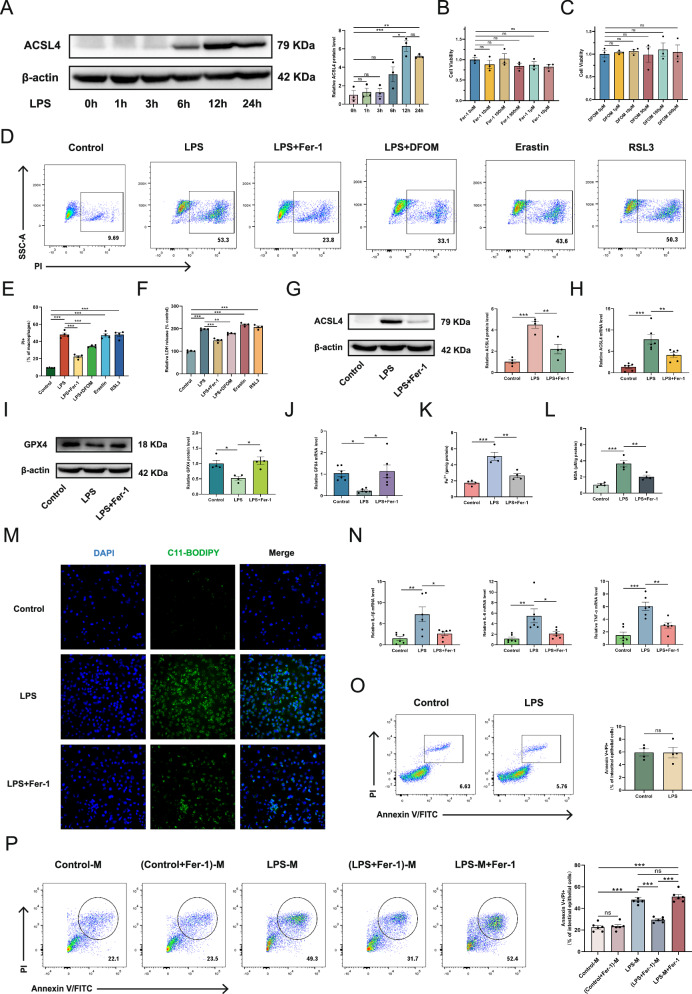
Inhibiting ferroptosis in inflammatory macrophages effectively alleviates intestinal epithelial cell death. **A** Western blot analysis of ACSL4 expression in RAW264.7 macrophages following LPS stimulation (1 μg/mL) at 0, 1, 3, 6, 12 and 24 h. The bar graph depicts the relative intensity of the bands. **B**, **C** The effect of different concentrations of Fer-1 and DFOM on the activity of RAW264.7 macrophages was assessed using the CCK-8 assay. **D**, **E** RAW264.7 macrophages were pre-treated separately with Fer-1 (1 μM), DFOM (100 μM), erastin (5 μM), and RSL3 (1 μM) for 30 min, followed by LPS stimulation (1 μg/mL) for 24 h. PI expression was detected by flow cytometry. **F** The release of LDH in the supernatant of macrophages in different treatment groups was detected. **G**–**J** RAW264.7 macrophages were pre-treated separately with Fer-1 (1 μM, 30 min), followed by LPS stimulation (1 μg/mL) for 24 h. Western blot and qpcr analysis of cellular expression of ACSL4 and GPX4 were conducted. **K**, **L** Determination of intracellular Fe²⁺ and MDA. **M** Detection of macrophage lipid peroxidation using the BODIPY 581/591 C11 probe. **N** IL-1β, IL-6, and TNF-α mRNA expression in RAW264.7 macrophages. **O**, **P** Representative flow cytometry plots for detecting intestinal epithelial cell death after different treatments. Data are mean ± SEM from 6 (**N**, **P**, **H**, **J**), 4 (**E**, **F**, **G**, **I**, **K**, **L**, **O**) and 3 (**A**, **B**, **C**). *p < 0.05, **p < 0.01, ***p < 0.001.

The above studies suggest that macrophage pyroptosis, apoptosis, and ferroptosis may play key roles in intestinal injury in NEC. We previously explored macrophage pyroptosis in NEC, confirming its role in inducing intestinal epithelial cell death [11, 12]. Additionally, some studies have explored apoptosis in NEC [19–21], but the role of macrophage ferroptosis remains unexplored. This section will examine the relationship between macrophage ferroptosis and intestinal epithelial cell injury. RAW264.7 macrophages were stimulated with LPS to establish an inflammatory model. To provide auxiliary guidance for LPS stimulation time selection, ACSL4 expression dynamics were assessed at multiple time points post-LPS exposure. Results at different time points of LPS stimulation showed that, compared to 0 h, ACSL4 significantly increased at 12 and 24 h (Fig. 5A). Prior to functional experiments, cytotoxicity assessment was performed to rule out confounding effects of the agents. RAW264.7 macrophages were exposed to escalating doses of Fer-1 (0–10 μM) or DFOM (0–200 μM) for 24 h. CCK-8 analysis revealed unaltered cell viability at all concentrations (*p* > 0.05 vs. vehicle control), confirming the absence of intrinsic cytotoxicity within the applied dosage windows (Fig. 5B, C). Ferroptosis is characterized by membrane rupture resulting from lipid peroxidation. Since propidium iodide (PI) uptake and lactate dehydrogenase (LDH) release directly reflect membrane integrity loss, a hallmark of cell death, we used these biomarkers to assess the LPS-induced macrophage death and the effects of ferroptosis-related drugs on it. Pharmacological perturbations using ferroptosis inhibitors (Fer-1, 1 μM; DFOM, 100 μM) and inducers (erastin, 5 μM; RSL3, 1 μM) revealed that LPS significantly increased PI+ macrophages versus controls (Fig. 5D, E), an effect substantially attenuated by Fer-1/DFOM but amplified by erastin/RSL3. Consistent LDH release patterns were observed (Fig. 5F), and validation in BMDMs confirmed reproducibility (Fig. S5). Collectively, these functional experiments demonstrate that ferroptosis contributes critically to LPS-triggered membrane rupture and macrophage death. Comparative assessment revealed that Fer-1 conferred superior protection against LPS-induced macrophage death relative to DFOM (Figs. 5D–F and S5). This efficacy advantage, coupled with Fer-1’s well-established specificity as a ferroptosis inhibitor, motivated its selection for subsequent mechanistic investigations into ferroptosis regulation. Fer-1 (1 μM, pretreated for 30 min) was applied to RAW264.7 cells, followed by LPS stimulation (1 μg/ml) for 24 h, and ferroptosis-associated parameters were subsequently assessed. The results revealed that Fer-1 significantly downregulated both protein and transcript levels of ACSL4, while concurrently upregulating GPX4 expression (Fig. 5G–J). Immunofluorescence showed that LPS stimulation increased ACSL4 expression (green) in macrophage cytoplasm, while Fer-1 treatment reduced the green fluorescence, indicating decreased ACSL4 levels (Fig. S6A). Compared to the control group, Fer-1 significantly inhibited the increase of Fe²⁺ in LPS-induced inflammatory macrophages (Fig. 5K). Additionally, Fer-1 also significantly reduced the increase of MDA levels induced by LPS (Fig. 5L). Using the BODIPY 581/591 C11 probe to detect lipid peroxidation in LPS-stimulated macrophages revealed that LPS increased lipid peroxidation levels (green) compared to the control group, while Fer-1 effectively reduced this activation in the cells (Fig. 5M). At the inflammatory level, Fer-1 markedly inhibited the elevated levels of IL-1β, IL-6, and TNF-α in LPS-induced inflammatory macrophages (Fig. 5N). These findings indicate that Fer-1 suppresses ferroptosis and attenuates inflammation in LPS-stimulated macrophages. Given the high LPS levels in NEC intestines, we examined its direct effect on intestinal epithelial injury. Stimulating IEC-6 cells with LPS (1 μg/ml, 24 h) revealed no significant cell damage compared to controls, confirming LPS alone does not markedly injure intestinal epithelial cells (Fig. 5O). To investigate macrophage ferroptosis’s role in driving inflammation and epithelial injury, we cultured intestinal epithelial cells for 24 h with supernatants from the following macrophage groups: control (control-M), control+Fer-1 ((control+Fer-1)-M), LPS-stimulated (LPS-M), and LPS+Fer-1 ((LPS+Fer-1)-M). Subsequent flow cytometry assessed epithelial cell damage. Additionally, to exclude any direct protective effect of Fer-1 on the intestinal epithelial cells themselves during the supernatant co-culture period, Fer-1 was added directly to epithelial cells cultured with LPS-M supernatant, establishing the LPS-M+Fer-1 group. Flow cytometry revealed no significant difference in epithelial cell damage between control-M and (control+Fer-1)-M groups (Fig. 5P). Compared with control-M, the LPS-M group exhibited significantly increased epithelial cell death, which was reduced by the (LPS+Fer-1)-M supernatant. However, direct Fer-1 addition to LPS-M supernatant (LPS-M+Fer-1 group) showed no protective effect. Immunofluorescence for the apoptosis protein cleaved caspase3 in intestinal epithelial cells across different treatment groups revealed consistent findings (Fig. S6B). These findings demonstrate that macrophage activation critically amplifies inflammatory injury, and targeting macrophage ferroptosis to modulate their inflammatory state can mitigate NEC-related intestinal injury.

### Inhibition of ferroptosis effectively improves intestinal injury in NEC mice

**Fig. 6:**
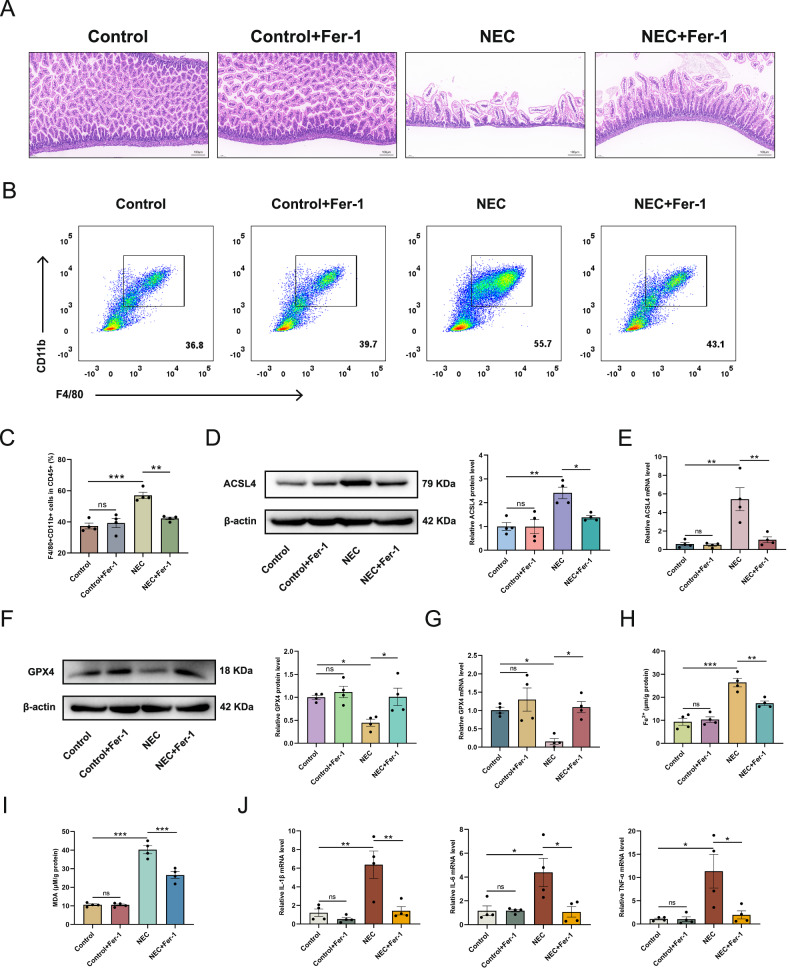
Inhibition of ferroptosis effectively improves intestinal injury in NEC mice. **A** Representative HE staining images of mice from different groups. **B** Flow cytometry results showing the percentage of macrophages among CD45+ cells in the intestinal tissues of mice from different groups. **C** The bar graph illustrates the intestinal macrophages in CD45+ cells in the different groups of mouse intestinal tissues. **D**–**G** Protein and mRNA expression levels of ACSL4 and GPX4 in the intestines of mice from different groups. **H**, **I** Determination of Fe²⁺ and MDA of intestinal tissues. **J** IL-1β, IL-6, and TNF-α mRNA expression in the intestines of mice from different groups. Data are mean ± SEM from 4 (**C**, **D**, **E**, **F**, **G**, **H**, **I**, **J**). *p < 0.05, **p < 0.01, ***p < 0.001.

In vitro experiments confirmed that inhibiting ferroptosis in inflammatory macrophages attenuates intestinal epithelial cell death. This finding was further validated in the NEC mouse model. Histological analysis revealed that Fer-1 administration restored intestinal villi architecture and mitigated tissue injury compared to NEC controls (Fig. 6A). Flow cytometry demonstrated reduced intestinal macrophage infiltration in Fer-1-treated NEC mice (Figs. 6B, C and S7). Molecular analyses indicated significantly elevated ACSL4 expression (mRNA/protein) with GPX4 downregulation in NEC versus controls, both reversed by Fer-1 treatment (Fig. 6D–G). Fer-1 also suppressed intestinal Fe²⁺ accumulation and MDA overproduction in NEC mice (Fig. 6H, I). Furthermore, Fer-1 attenuated the NEC-induced upregulation of proinflammatory cytokines (IL-1β, IL-6, TNF-α) (Fig. 6J). Collectively, Fer-1 inhibits intestinal ferroptosis in NEC, thereby reducing inflammation and macrophage infiltration.

## Discussion

In this study, we explored the changes of macrophages in NEC by using scRNA-seq analysis, bulk RNA-seq analysis, patient clinical sample detection, in vivo and in vitro experiments, and focused on the effect of macrophage ferroptosis on intestinal epithelial cell injury. The main findings are as follows: (1) significant infiltration of macrophages is observed in the intestines of NEC; (2) scRNA-seq analysis identified the predominant macrophage population in NEC as proinflammatory macrophages; (3) the predominant macrophage population in NEC was characterized as highly prone to cell death (apoptosis, pyroptosis, and ferroptosis); (4) Inhibition of ferroptosis in inflammatory macrophages effectively reduced the death of intestinal epithelial cells; (5) The ferroptosis inhibitor Fer-1 significantly alleviated intestinal inflammation and improved intestinal damage in NEC mice. In conclusion, targeting and modulating macrophage ferroptosis may serve as an important therapeutic strategy for NEC.

NEC is a life-threatening and devastating acute gastrointestinal disease in neonates. To date, the exact etiology and pathogenesis of NEC remain unclear. Factors such as enteral feeding, bacterial infections, dysbiosis, inflammatory responses, hypoxia, and ischemia-reperfusion injury are closely associated with the onset and progression of NEC [22]. Currently, the main treatment strategies for NEC include parenteral nutrition, broad-spectrum antibiotics, and surgical intervention. However, these treatments have limited efficacy and are often accompanied by complications such as secondary infections, gut microbiota disruption, and short bowel syndrome [23]. With the rapid development of molecular biology and immunology, there has been a deeper understanding of immune dysregulation in NEC. Dysbiosis in the gut triggers sustained activation of immune cells and the release of cytokines in the immature immune system of neonates, which leads to an inflammatory storm, resulting in epithelial cell death and ongoing damage to the intestinal wall [24]. Numerous studies have shown a close relationship between the chemotactic recruitment and inflammatory activation of macrophages and the intestinal damage caused by NEC [25, 26]. In this study, omics technologies and experimental data revealed significant macrophage infiltration in the intestines of both NEC patients and the mouse model, with these infiltrating macrophages exhibiting high proinflammatory activity and chemotaxis. Moreover, LPS alone does not cause significant damage to intestinal epithelial cells, whereas the supernatant from LPS-stimulated macrophages induces substantial cell death in intestinal epithelial cells. These findings highlight the amplifying and cascading role of macrophage inflammatory activation in intestinal epithelial injury. Therefore, modulating the overactivation of the immune system and inhibiting the excessive activation of macrophages may offer a promising strategy to mitigate intestinal damage during NEC progression.

The emergence of single-cell sequencing technology has been revolutionary. Compared to traditional tissue transcriptome sequencing, it allows researchers to focus on the changes in specific cell populations. In this study, we further categorized macrophages into subgroups and discovered significant differences in the characteristics of the main macrophage populations between the NEC group and the control group. We focused on the main macrophage population in the NEC group and performed enrichment analysis on the top marker genes. The results indicated that these macrophages were closely associated with various cell death pathways. After collecting genes related to cell death, we constructed gene sets and scored them in the single-cell data. The results showed that the main macrophage population in the NEC group exhibited high apoptosis, pyroptosis, and ferroptosis characteristics, and there was a significant positive correlation with the proinflammatory score. Immunofluorescence experiments and pseudotime analysis further validated these results. Additionally, some studies have experimentally explored pyroptosis and apoptosis in macrophages in NEC, and these results align with our findings. However, there is a lack of research on ferroptosis in macrophages in NEC. Therefore, we focused on examining the changes in ferroptosis of macrophages in NEC.

Ferroptosis is a regulated form of cell death characterized by iron-dependent, uncontrolled accumulation of lipid peroxides, ultimately leading to oxidative damage and collapse of cellular membrane systems [27]. Its core mechanism involves disruption of intracellular redox homeostasis, particularly dysfunction of glutathione peroxidase 4 (GPX4). As a key lipid-repair enzyme, GPX4 utilizes reduced glutathione to convert toxic lipid hydroperoxides in membrane phospholipids into nontoxic lipid alcohols, thereby maintaining membrane lipid homeostasis [28, 29]. Ferroptosis is critically dependent on accumulation of intracellular labile iron ions (Fe²⁺). Excess Fe²⁺ drives the lipid peroxidation cascade via the Fenton reaction or activation of lipoxygenases (LOXs), amplifying oxidative damage. Furthermore, acyl-CoA synthetase long-chain family member 4 (ACSL4) potentiates ferroptosis by activating long-chain PUFAs and facilitating their incorporation into membrane phospholipids, thereby increasing membrane susceptibility to peroxidation [30, 31]. Elevated ACSL4 expression or activity accelerates ferroptotic death. Uncontrolled lipid peroxidation generates toxic terminal products such as malondialdehyde (MDA) and 4-hydroxynonenal (4HNE), which disrupt membrane integrity and trigger cellular disintegration. Recent studies have shown that ferroptosis plays a critical role in various cancers and inflammatory diseases [32, 33]. Inhibition of ferroptosis in macrophages has demonstrated promising therapeutic effects in several diseases [34, 35]. Analysis of the single-cell sequencing data revealed aberrant activation of ferroptosis in macrophages during NEC. Clinical evidence revealed elevated expression of ACSL4 and 4HNE in intestinal macrophages from NEC patients, suggesting dysregulated ferroptotic cell death. This phenomenon was corroborated in vitro: LPS stimulation induced profound macrophage membrane rupture and cell death, which was effectively rescued by the ferroptosis inhibitor. Mechanistically, LPS triggered a cascade of ferroptosis-promoting events, including ACSL4 upregulation, GPX4 suppression, intracellular Fe²⁺ accumulation, and elevated lipid peroxidation, concomitant with enhanced proinflammatory cytokine expression. Fer-1 treatment significantly attenuated all these pathological alterations, confirming ferroptosis as the central driver of LPS-induced macrophage death. Furthermore, supernatants from Fer-1-treated, LPS-stimulated macrophages significantly attenuated intestinal epithelial cell death under inflammatory conditions. Conversely, direct addition of Fer-1 to IECs failed to rescue cell death when cultured with supernatants from LPS-activated macrophages, underscoring that the critical role of macrophage ferroptosis in intestinal injury. This also reveals the importance of regulating immune system dysregulation to improve intestinal damage. Fer-1 also significantly reduced the expression of inflammatory factors in the intestines of NEC mice and improved intestinal inflammatory injury. These findings suggest that Fer-1 may exert anti-inflammatory protective effects in NEC by inhibiting macrophage ferroptosis, offering a new perspective for NEC research. Notably, emerging research proposes a paradoxical role for ferroptosis in in vivo inflammation. Davidson AJ et al. demonstrated that while ferroptosis facilitates immune cell recruitment during wound healing and activates tissue-intrinsic antimicrobial defenses, it simultaneously poses inherent challenges for macrophage clearance [36]. This leads to frustrated phagocytosis and frequent corpse fragmentation. The dysregulated cytotoxic pathways activated during ferroptosis may exceed the scope of evolutionarily beneficial outcomes. Furthermore, impaired macrophage efferocytosis can hinder antigen presentation, and ferroptosis itself might actively suppress antigen presentation, thereby impeding the activation of adaptive immunity. Kim R et al. discovered that in the tumor microenvironment (TME), ferroptosis in pathologically activated neutrophils (PMN), termed myeloid-derived suppressor cells (PMN-MDSCs), induces the release of oxidized lipids that suppress the activity of both murine and human T cells [37]. Inhibiting ferroptosis alleviates this immunosuppression, delays tumor growth, and synergizes with immune checkpoint blockade (ICB). These recent findings challenge the conventional paradigm surrounding ferroptosis. Consequently, therapeutically targeting ferroptosis within inflammatory milieus holds potential for mitigating inflammatory damage; however, this strategy requires careful risk-benefit assessment due to the possibility of compromising early immune responses. Similarly, inhibiting ferroptosis may emerge as a novel strategy to improve responses to immunotherapy, but future research must prioritize the development of cell-type-specific targeting approaches.

There are some limitations in this study. First, the experimental part of this study utilized the LPS-induced inflammatory macrophage model and NEC mouse model, which may not fully replicate the complexity of human NEC. Therefore, the clinical applicability of the findings requires further validation. Second, although this study revealed the role of macrophage ferroptosis in NEC-induced intestinal damage, the specific targets and mechanisms for regulating macrophage ferroptosis during NEC progression have not been thoroughly investigated. Third, while this study focuses on regulating macrophage inflammatory activation to alleviate intestinal epithelial cell damage, the mechanisms of cell death in intestinal epithelial cells themselves remain unclear and warrant further exploration in future research. For macrophages, this study focuses on classic cell death modalities, including apoptosis, ferroptosis, pyroptosis, and necroptosis. Recent research has introduced novel forms of cell death, such as cuproptosis, disulfidptosis, and natroptosis, which warrant further investigation in the context of NEC in the future. Moreover, this study utilized C57BL/6J substrain mice. This substrain exhibits genotypic and phenotypic distinctions from C57BL/6N. Currently, no systematic investigations link substrain differences to ferroptosis susceptibility, including within NEC models—an important avenue for future research. Finally, regarding the dysregulated immune system in NEC, this study primarily focused on macrophages, while the activation and roles of other immune cells in NEC require further investigation.

Overall, this study combines omics techniques and experimental research to thoroughly investigate the changes in macrophages in NEC. It also, for the first time, reveals that inhibiting ferroptosis in macrophages can effectively reduce inflammatory infiltration and improve intestinal epithelial damage. These findings provide new evidence for the molecular mechanisms underlying NEC-related intestinal injury and support macrophage ferroptosis as a potential therapeutic target for NEC.

## Materials and methods

### Data sources and processing

In this study, single-cell raw data provided by Egozi et al. were utilized for single-cell analysis (available at 10.5281/zenodo.5813397) [38]. To validate the conclusions derived from the aforementioned single-cell dataset, the GSE178088 single-cell dataset from the Gene Expression Omnibus (GEO) database (accessible at https://www.ncbi.nlm.nih.gov/) was employed [39]. Additionally, GSE46619 and GSE64801 datasets were used as bulk RNA-seq data for NEC to perform tissue-level transcriptomic sequencing analysis [40–42].

### Analysis of scRNA-seq data

The scRNA-seq analysis was performed systematically following these steps: (1) Data format conversion: Prepare the 10×scRNA-seq data and convert it into a Seurat object using the “CreateSeuratObject” function from the R package “Seurat” [43]; (2) Quality control (QC): Filter data by retaining cells with a mitochondrial gene RNA percentage (percent.mt) less than 40%, a total detected RNA count (nCount_RNA) greater than 1000, and a detected gene count (nFeature_RNA) greater than 300, thus excluding low-quality cells; (3) Normalization and standardization: Normalize and standardize the QC-filtered data using the “NormalizeData” and “ScaleData” functions, and identify the top 2000 highly variable genes using the “FindVariableFeatures” function; (4) Dimensionality reduction and clustering: Perform principal component analysis (PCA) using the “RunPCA” function, and apply uniform manifold approximation and projection (UMAP) for dimensionality reduction and clustering [44]; (5) Cell annotation: Set the parameters min.pct and logfc.threshold to 0.25, and use the “FindAllMarkers” function to identify top marker genes for each cluster. Annotate the clusters using the CellMarker 2.0 database (http://117.50.127.228/CellMarker/) and the Cell Taxonomy database (http://ngdc.cncb.ac.cn/CellTaxonomy/) [45, 46]; (6) Macrophage reclustering: Extract macrophage data from seurat data using the “subset” function and apply the same steps of dimensionality reduction and clustering as in the previous steps. Perform enrichment analysis of the top marker genes of the predominant macrophage population in the NEC cohort using the Metascape database (https://Metascape.org/) [47]; (7) Gene set scoring: Retrieve genes associated with pro-inflammation, chemotaxis, apoptosis, necroptosis, ferroptosis, and pyroptosis from the GeneCards database (https://www.genecards.org/) and the KEGG database (https://www.genome.jp/kegg/) (Supplementary material 1–6) [48–50]. Use the PercentageFeatureSet scoring method to compare scores across different groups and macrophage clusters. Visualize the results using the “ggviolin” function for violin plots, the “FeaturePlot” function to display scores on UMAP plot, and the “ggscatterstats” function to plot correlations between different death and inflammation pathways; (8) Pseudotime Analysis: Construct a monocle object using the R package “monocle” and the “newCellDataSet” function [51]. Perform dimensionality reduction using the “DDRTree” method, and calculate cell pseudotime with the “orderCells” function. Analyze pseudotime-ordered cell data and specified nodes using Branched Expression Analysis Modeling (BEAM) to identify differential genes at branch points and perform enrichment analysis.

### Analysis of immune infiltration

A batch correction was performed on the merged bulk RNA-seq datasets GSE46619 and GSE64801 using the “ComBat” function from the “sva” package. To evaluate the proportion of infiltrating immune cells, single-sample gene set enrichment analysis (ssGSEA) was conducted using the “GSVA” package [52]. The data were converted into matrix format, with the method set to “ssgsea” and kcdf set to “Gaussian”, followed by analysis using the “gsva” function. Immune infiltration was visualized using box plots generated by ggplot.

### Human clinical samples

This study will use intestinal samples obtained from NEC and intestinal atresia (IA) patients who underwent surgery at the Zhejiang University School of Medicine Affiliated Children’s Hospital. Based on gross morphology, necrotic intestinal tissue from NEC patients and non-inflammatory intestinal tissue from IA patients will be collected and sent to the pathology department for paraffin embedding and sectioning. Intestinal tissue from IA patients with normal histology and no inflammatory damage will serve as the normal control group for the experiments.

### Experimental animals

The C57BL/6J mice used in this study were purchased from the Shanghai Slac Laboratory Animal Company (Shanghai, China). Mice approximately 5 days old and weighing around 2.0 g were selected for the experiments. All mice were housed in a pathogen-free environment with a regular light-dark cycle. Animal experiments and procedures were conducted in accordance with the Guide for the Care and Use of Medical Laboratory Animals stipulated by the Ministry of Health of China, and approved by the Institutional Animal Care and Use Committee of Zhejiang University, Zhejiang Province, China. The number of experimental animals used was determined based on the laboratory’s prior experience, results from preliminary experiments, and similar studies on the disease, in accordance with the 3 R (Replacement, Reduction, Refinement) principles approved by the Animal Ethics Committee [11, 12].

### Induction and treatment of NEC

Mice were numbered and randomly assigned to the control group and NEC group using a computer-generated random number method. In the control group, newborn mice were naturally breastfed. In the NEC group, mice were gavaged with 30% Esbilac formula milk (Pet-Ag, New Hampshire, IL, USA) using a 5 cm curved gavage tube. Based on preliminary modeling experience and pilot studies, mice were administered 40–60 μl of formula milk via oral gavage at 4-h intervals. At the same time, NEC group mice were subjected to hypoxia (99.9% N_2_, lasting 90 s) and cold stress (4 °C, 10 min), twice daily (morning and evening). In the control group, half of the mice additionally received intraperitoneal injections of ferroptosis inhibitor Ferrostatin-1 (Fer-1) (5 mg/kg, HY-100579, MedChem Express, Monmouth Junction, NJ, USA) to eliminate potential drug effects (control+Fer-1 group). In the NEC group, half of the mice received intraperitoneal injections of Fer-1 (5 mg/kg) (NEC+Fer-1 group). On day 5, all mice were euthanized, and intestinal samples were collected for subsequent experiments.

### Hematoxylin and eosin staining

The collected intestinal tissues were placed in 4% paraformaldehyde and subsequently embedded in paraffin. After embedding, tissue sections were prepared and stained with hematoxylin and eosin (H&E), and then examined under an Olympus optical microscope. Two senior pathologists assessed the intestinal tissue damage using a double-blind method. The damage scoring criteria were as follows: (1) Normal tissue structure, intact villi and epithelium: 0 points; (2) Mild separation of the submucosa and/or lamina propria: 1 point; (3) Moderate separation of the submucosa and/or lamina propria, and/or edema of the submucosa and muscle layer: 2 points; (4) Severe separation of the submucosa and/or lamina propria, and/or edema of the submucosa and muscle layer, with partial villus shedding: 3 points; (5) Complete villus disappearance with intestinal necrosis: 4 points [53]. Tissues with a score of 2 or higher were considered as NEC intestinal injury models.

### Cell culture and treatment

The RAW264.7 macrophage cell line was obtained from the American Type Culture Collection (ATCC), and the IEC-6 intestinal epithelial cell line was sourced from Cell Bank (Shanghai, China). All cell lines were authenticated by short tandem repeat (STR) profiling within the last 6 months and confirmed mycoplasma-free using the MycoAlert® Mycoplasma Detection Kit (Lonza) prior to experimentation. Both cell types were cultured in Dulbecco’s modified Eagle’s medium (DMEM) (Gibco, Grand Island, NY, USA) containing 10% fetal bovine serum (Ausgenex, Australia) and 1% penicillin/streptomycin (Sangon Biotech, Shanghai, China) in a 37 °C, 5% CO_2_ incubator. LPS (L2654, Sigma-Aldrich) was dissolved in PBS or DMEM according to the manufacturer’s instructions, while Fer-1 (MedChem Express), Deferoxamine Mesylate (DFOM) (T1637, TOPSCIENCE, Shanghai, China), erastin (T1765, TOPSCIENCE) and RSL3 (T3646, TOPSCIENCE) were dissolved in DMSO (Sigma-Aldrich). Cells were seeded at a density of 3–5 × 10^5^ per well in six-well plates. Following 24 h adhesion, cells were stimulated with LPS (1 μg/mL) for 24 h in the presence of either the ferroptosis inhibitor Fer-1 (1 μM) or the iron chelator DFOM (100 μM). Separately, ferroptosis inducers erastin (5 μM) or RSL3 (1 μM) were administered without LPS to induce ferroptosis. To study the effect of macrophage on intestinal epithelial cell damage, after 24 h of culturing the intestinal epithelial cells, the supernatant was discarded, and intestinal epithelial cells were washed with PBS. The supernatant from treated macrophages was then added to the epithelial cells, and after 24 h of incubation, subsequent assays were performed.

### Extraction of bone marrow-derived macrophages

Bone marrow-derived macrophages (BMDMs) were isolated from femurs and tibias of 6-8-week-old C57BL/6 mice. After euthanasia, mice were surface-sterilized with 75% ethanol for 5 min. The tibia and femur were obtained using sterile instruments and gauze, washed with PBS, and centrifuged (400 × *g*, 5 min). After the supernatant was discarded, red blood cell lysate was added to the precipitate for lysis, and then terminated with PBS and centrifuged (400 × g, 5 min). Cell pellets were resuspended in complete DMEM (10% FBS, 1% penicillin/streptomycin, 50 ng/ml M-CSF) and plated. After 24 h, non-adherent cells were transferred to new plates. On day 4, medium was replaced. Adherent macrophages were harvested by gentle scraping on day 7 for subsequent experiments.

### Flow cytometry

The isolation of mouse intestinal macrophages was performed based on relevant literature and our laboratory’s previous experience [54]. Briefly, after washing the small intestine with PBS, the tissue was cut into small pieces and immersed in Hanks’ Balanced Salt Solution (HBSS) (Absin) containing 5 mM ethylenediaminetetraacetic acid (EDTA) (Sigma-Aldrich, St. Louis, MO, USA) and 1 mM dithiothreitol (DTT) (Sigma-Aldrich), then incubated in a 37 °C orbital shaker for 30 min. The intestinal tissue was then removed, further minced, and transferred to DMEM containing 5% bovine serum albumin (BSA) (Meilunbio, Dalian, China) and 75 µg/mL Liberase TM (Sigma-Aldrich). The shaking step was repeated. After light centrifugation, the supernatant was collected and further centrifuged (350 g, 5 min). The pellet was washed twice with PBS and then stained with Fixable Viability Stain 510 (FVS510, 564406, BD Biosciences, San Jose, CA, USA), CD45 (557659, BD Biosciences), F4/80 (565411, BD), and CD11b (53-012-82, Invitrogen) in the dark for 15 min. Flow cytometry was performed using the FACSLyric™ flow cytometer (663518, BD), and results were analyzed with FlowJo software (Version X; TreeStar, Ashland, OR, USA). Death of macropahges and intestinal epithelial cells were detected using the BD Pharmingen™ FITC Annexin V Apoptosis Detection Kit I (566547, BD), following the kit’s instructions strictly.

### Immunohistochemical staining

Paraffin sections of the intestinal tissue were deparaffinized and rehydrated, followed by antigen retrieval. After washing, the sections were incubated with 5% BSA for 1 h to block nonspecific binding. After blocking, CD68 (sc20060, Santa Cruz Biotechnology, Santa Cruz, CA, USA) or CD68 (14-0681-82, Invitrogen, Carlsbad, CA, USA) (1:100) was added, and the sections were incubated overnight at 4 °C. The following day, horseradish peroxidase-conjugated secondary antibodies (Beyotime, Shanghai, China) were applied at room temperature for 1 h. Finally, the sections were stained with DAB solution (Sangon Biotech) and hematoxylin. Immunohistochemical results were observed under the optical microscope.

### Immunofluorescence staining

The steps for immunofluorescence are similar to immunohistochemistry. After deparaffinization, rehydration, antigen retrieval, and blocking, the sections were incubated overnight at 4 °C with CD68, ACSL4 (22401-1-AP, Proteintech), 4-Hydroxynonenal (4HNE) (PC6313, Abmart, Shanghai, China) and Cleaved Caspase3 (25128-1-AP, Proteintech) (1:50). After incubation, Alexa Fluor 488-labeled and 594-labeled secondary antibodies (111-545-144 and 115-585-146, Jackson Immunoresearch Laboratories, West Grove, PA, USA) were applied and incubated in the dark for 30 min. Finally, DAPI Stain Solution (Sangon Biotech) was added for nuclear staining. The samples were then examined under the Zeiss microscope (Carl Zeiss, Jena, Germany). The procedure for cellular immunofluorescence is similar to tissue immunofluorescence. Briefly, cells were plated onto coverslips and subjected to the appropriate treatments. After fixation, permeabilization, and blocking, the cells were incubated overnight at 4 °C with primary antibodies. The next day, after washing, the cellular slides were incubated with the appropriate secondary antibodies at room temperature in the dark for 30 min. After further washing, the cellular slides were mounted with an anti-fade reagent containing DAPI and observed under the fluorescence microscope.

### Detection of Lactate dehydrogenase

Plasma membrane rupture during cell death releases cytoplasmic enzymes into the culture medium, including stable lactate dehydrogenase (LDH). Quantifying LDH activity in the supernatant serves as a sensitive indicator of membrane integrity and enables cytotoxicity assessment. We collected supernatants from treated macrophages and measured LDH release using LDH Cytotoxicity Assay Kit (C0019S, Beyotime) per manufacturer’s protocol.

### Western blot

The protein extraction procedure for cells and tissues is similar. Briefly, cells or tissue samples were lysed in radioimmunoprecipitation assay (RIPA) buffer (Boster, Wuhan, China) containing 1% phosphatase inhibitors and a protease inhibitor cocktail (Sigma-Aldrich) for 30 min. The samples were then centrifuged (12,000 × *g*, 10 min, 4 °C). Tissue samples added to the lysis buffer required homogenization. The supernatant was collected, and protein concentration was determined using a BCA protein assay kit (Meilunbio, Dalian, China). The appropriate amount of supernatant was mixed with 5×SDS/PAGE sample buffer (Beyotime) for concentration normalization. Proteins were separated on an SDS/PAGE gel and then transferred to a PVDF membrane (Sigma-Aldrich). After blocking with 5% BSA for 1 h, the membrane was incubated overnight at 4 °C with ACSL4 (22401-1-AP, Proteintech), GPX4 (67763-1-Ig, Proteintech), and Beta Actin (66009-1-Ig, Proteintech) (1:1000) on a shaking platform. The next day, the membrane was incubated with the corresponding secondary antibody (Proteintech) for 1 h. Following this, a hypersensitive ECL chemiluminescence solution (Thermo Fisher Scientific) was applied to the membrane, and imaging was performed using the GeneSys Chemi imaging system (Syngene G: BOX, USA). The acquired bands were quantified using ImageJ software.

### Cell counting Kit-8 assay

Approximately 5000 cells per well were seeded in a 96-well plate. After treatment with different concentrations of Fer-1 and DFOM, 100 μl of culture medium and 10 μl of cell counting kit-8 (CCK-8) solution (Beyotime) were added to each well and incubated at 37 °C for 1.5–2 h. The absorbance at 450 nm was measured and collected from each well for subsequent analysis.

### Quantitative real-time PCR

RNA was extracted using TRIzol, chloroform, and the SteadyPure RNA Extraction Kit (Accurate Biology, Changsha, China). Reverse transcription of RNA was performed using PrimeScript™ RT Master Mix (Takara Bio Inc., Otsu, Shiga, Japan). Quantitative real-time PCR (qRT-PCR) for each gene was conducted using TB Green® Premix Ex Taq™ II (Takara Bio Inc., Otsu, Shiga, Japan). All steps were strictly followed according to the kit instructions. β-actin was used as the baseline control gene, and the relative mRNA levels were calculated using the 2^−ΔΔCt^ method. The primers used were designed on the PrimeBank website (https://PGA.mgh.Harvard.edu/primerbank/), and the specific sequences are shown as follows (Forward primer in front, Reverse primer in the back):

1. Il-1β: GCAACTGTTCCTGAACTCAACT, ATCTTTTGGGGTCCGTCAACT;
2. Il-6: TAGTCCTTCCTACCCCAATTTCC, TTGGTCCTTAGCCACTCCTTC;
3. Tnf-α: CCCTCACACTCAGATCATCTTCT, GCTACGACGTGGGCTACAG;
4. β-actin: GGCTGTATTCCCCTCCATCG, CCAGTTGGTAACAATGCCATGT;
5. Acsl4: CTCACCATTATATTGCTGCCTGT, TCTCTTTGCCATAGCGTTTTTCT;
6. Gpx4: GATGGAGCCCATTCCTGAACC, CCCTGTACTTATCCAGGCAGA.

### Fe^2+^, MDA and lipid peroxidation measurements

Free divalent iron ions (Fe²⁺) can induce spontaneous lipid peroxidation via the Fenton reaction. The resulting reactive oxygen species (ROS) cause damage to cell membrane structures. Therefore, the detection of iron overload and lipid peroxidation are commonly used as important indicators for assessing ferroptosis. Intestinal tissue or RAW264.7 cells were collected and Fe^2+^ levels were measured using the Fe^2+^ assay kit (Elabscience, Wuhan, China). Lipid peroxidation was assessed using the MDA assay kit (Beyotime, Shanghai, China) and Lipid peroxidation detection kit (BODIPY 581/591 C11) (Beyotime, Shanghai, China). High levels of intracellular malondialdehyde (MDA) indicate lipid peroxidation. BODIPY 581/591 C11 is a lipid peroxidation probe that rapidly enters cell membranes and is used to detect lipid peroxidation (green fluorescence) and antioxidant status (red fluorescence) in live cells. The procedures were performed according to the instructions provided in the respective kits.

### Statistical analysis

All experiments were performed independently at least three times. Data are presented as the mean ± SEM. Statistical analysis of the experimental results was performed using SPSS 21.0 software (IBM Corporation, Armonk, NY, USA). When data showed normal distribution and homogeneity of variance, comparisons between two groups were conducted using independent sample t-tests, while comparisons among multiple groups were performed using one-way analysis of variance (ANOVA) followed by Bonferroni post-hoc tests. A *p*-value < 0.05 was considered statistically significant. Bar charts representing the experimental results and statistical analysis were generated using GraphPad Prism 7.0 software (GraphPad Software, San Diego, CA, USA).

## Supplementary information

Supplementary Figures

Original blots

Supplementary material 1 proinflammatory genes

Supplementary material 2 chemokines genes

Supplementary material 3 apoptosis genes

Supplementary material 4 pyroptosis genes

Supplementary material 5 ferroptosis genes

Supplementary material 6 necroptosis genes

## Data Availability

The single-cell raw data provided by Egozi A et al. are available for single-cell analysis (available at 10.5281/zenodo.5813397). The other datasets analyzed during the current study are available in the GEO repository, https://www.ncbi.nlm.nih.gov/.
