# Supplementary material for: Inhibition of ferroptosis in inflammatory macrophages alleviates intestinal injury in neonatal necrotizing enterocolitis
Source: Cell Death Discov. 2025 Aug 5;11:365. doi: 10.1038/s41420-025-02665-9 (PMC12325787; doi:10.1038/s41420-025-02665-9)
Supplement: Supplementary file 1 — Supplementary Figures [file 41420_2025_2665_MOESM1_ESM.docx]

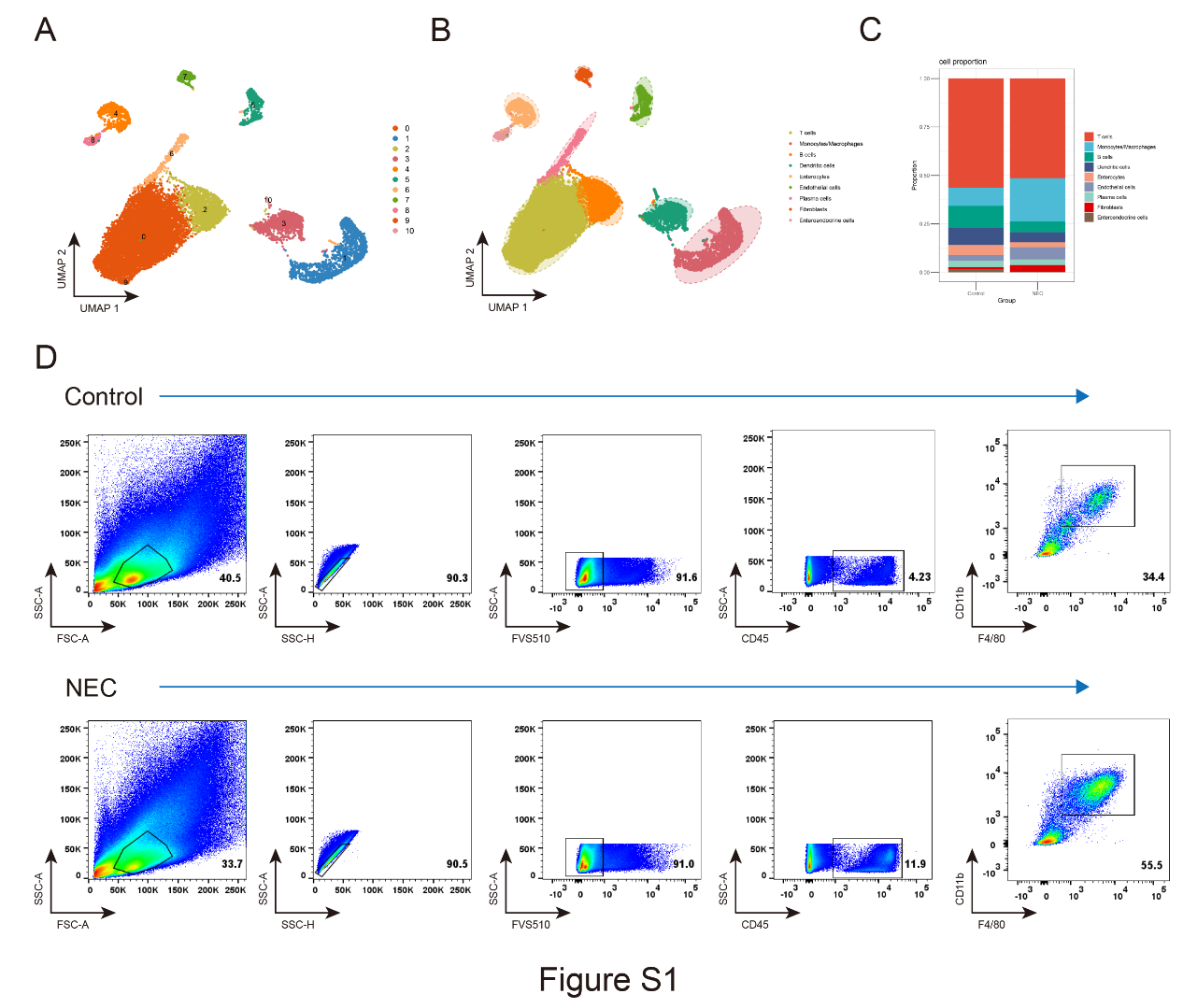


**Figure S1** Validation analysis of the scRNA-seq dataset GSE178088 and gating strategy for mouse intestinal flow cytometry. (**A-B**) UMAP dimensionality reduction identified 11 cell clusters, which were annotated into 9 distinct cell types. (**C**) Stacked bar plot showing the cellular composition. (**D**) Gating strategy schematic for mouse intestinal flow cytometry.


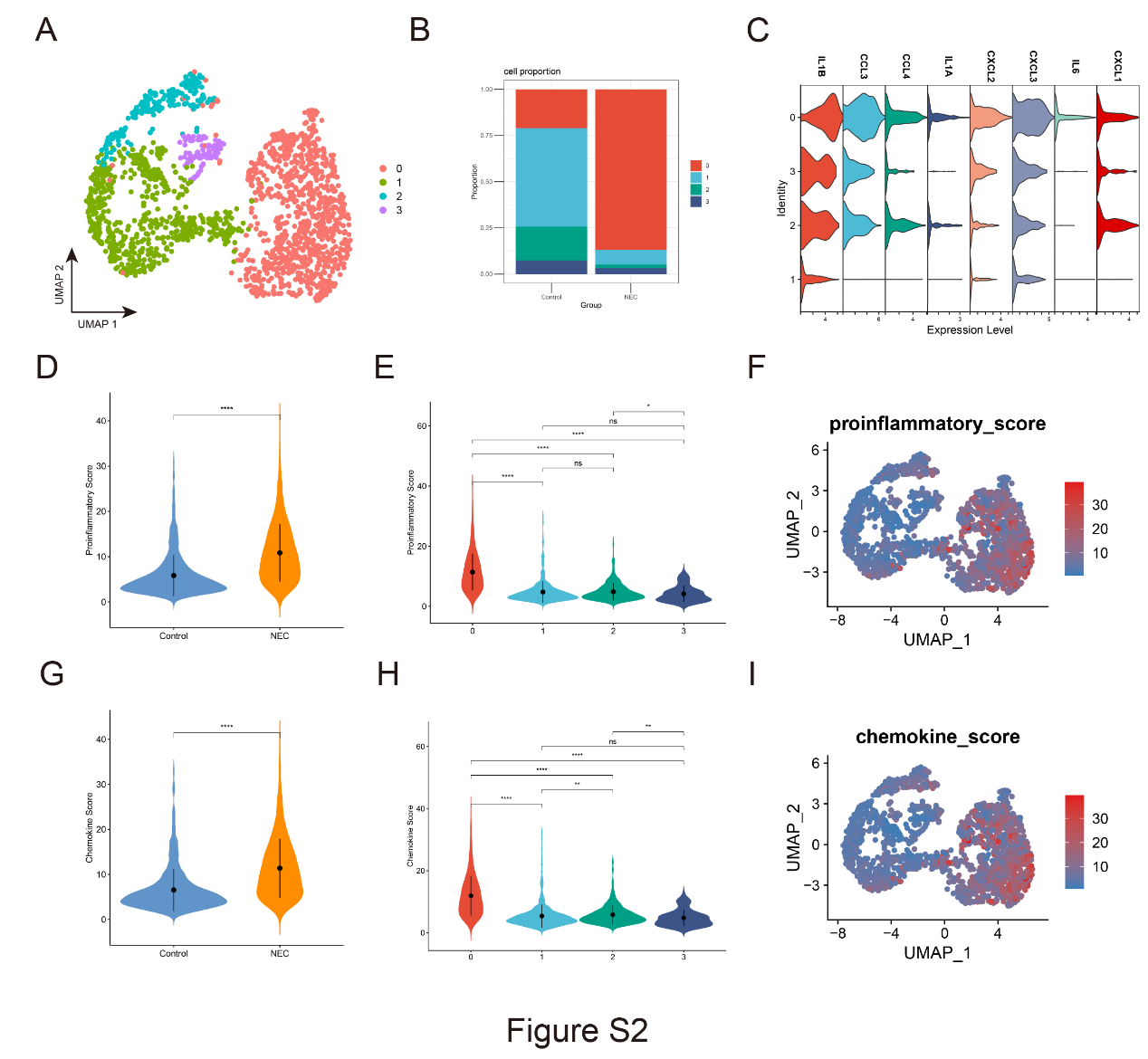


**Figure S2** Validation of proinflammatory and chemokine signatures in the scRNA-seq dataset GSE178088. (**A**) Reclustering of macrophages identified four distinct subtypes. (**B**) Stacked bar plot depicting the proportions of macrophage subtypes. (**C**) Violin plots showing expression levels of proinflammatory factors and chemokines across macrophage subpopulations. (**D**) Violin plots comparing proinflammatory scores in macrophages between control and NEC groups. (**E**) Violin plots displaying proinflammatory scores across macrophage subpopulations. (**F**) UMAP visualization of proinflammatory scores. (**G**) Violin plots comparing chemotactic scores in macrophages between control and NEC groups. (**H**) Violin plots showing chemokine scores across macrophage subpopulations. (**I**) UMAP visualization of chemokine scores.


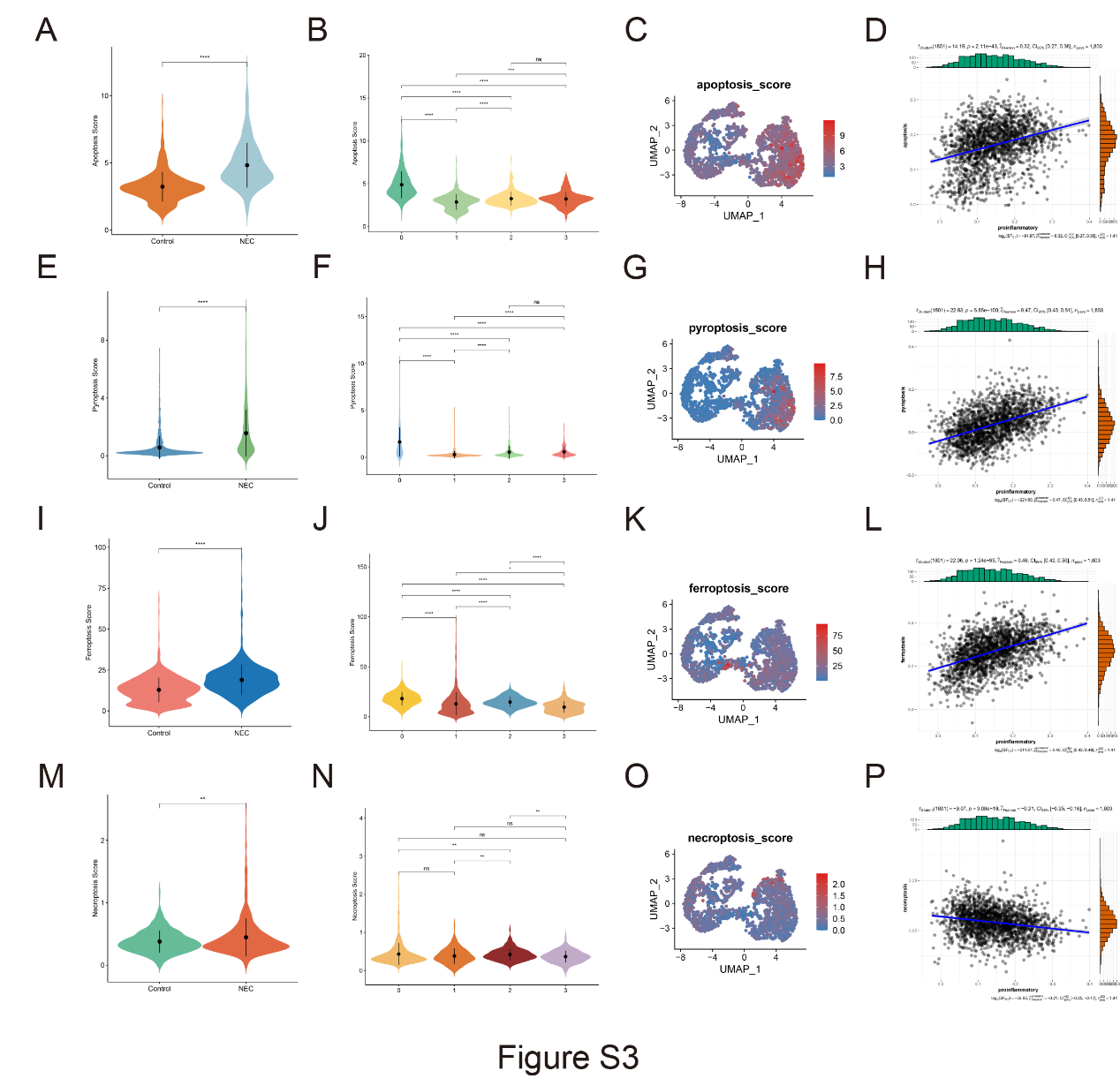


**Figure S3** Validation analysis of apoptosis, pyroptosis, ferroptosis, and necroptosis in the scRNA-seq dataset GSE178088. (**A, E, I, M**) Violin plots comparing apoptosis, pyroptosis, ferroptosis, and necroptosis signature scores in macrophages between NEC and control groups. (**B, F, J, N**) Violin plots displaying apoptosis, pyroptosis, ferroptosis, and necroptosis scores across macrophage subpopulations. (**C, G, K, O**) UMAP visualization of apoptosis, pyroptosis, ferroptosis, and necroptosis scores of macrophage subpopulations. (**D, H, L, P**) Scatter plots showing correlation analyses between proinflammatory scores and apoptosis/pyroptosis/ferroptosis/necroptosis scores.


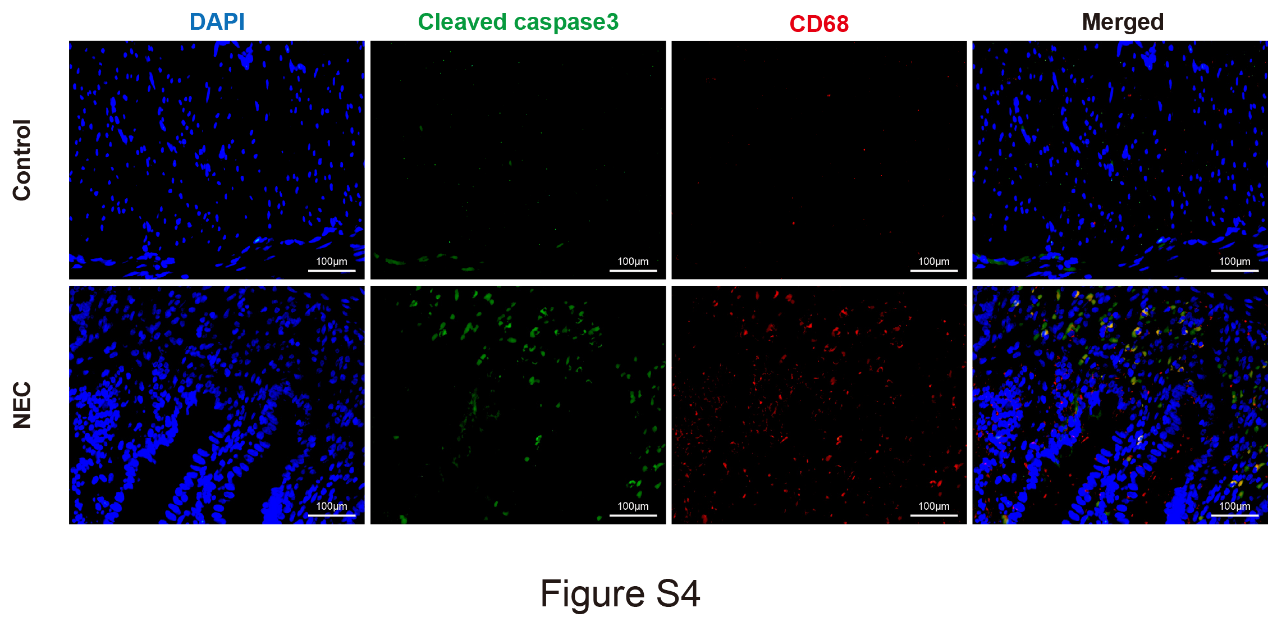


**Figure S4** Immunofluorescence staining shows the co-localization of cleaved caspase3 and CD68 positive cells in intestinal tissue samples from NEC and control patients (scale bar: 100 µm).


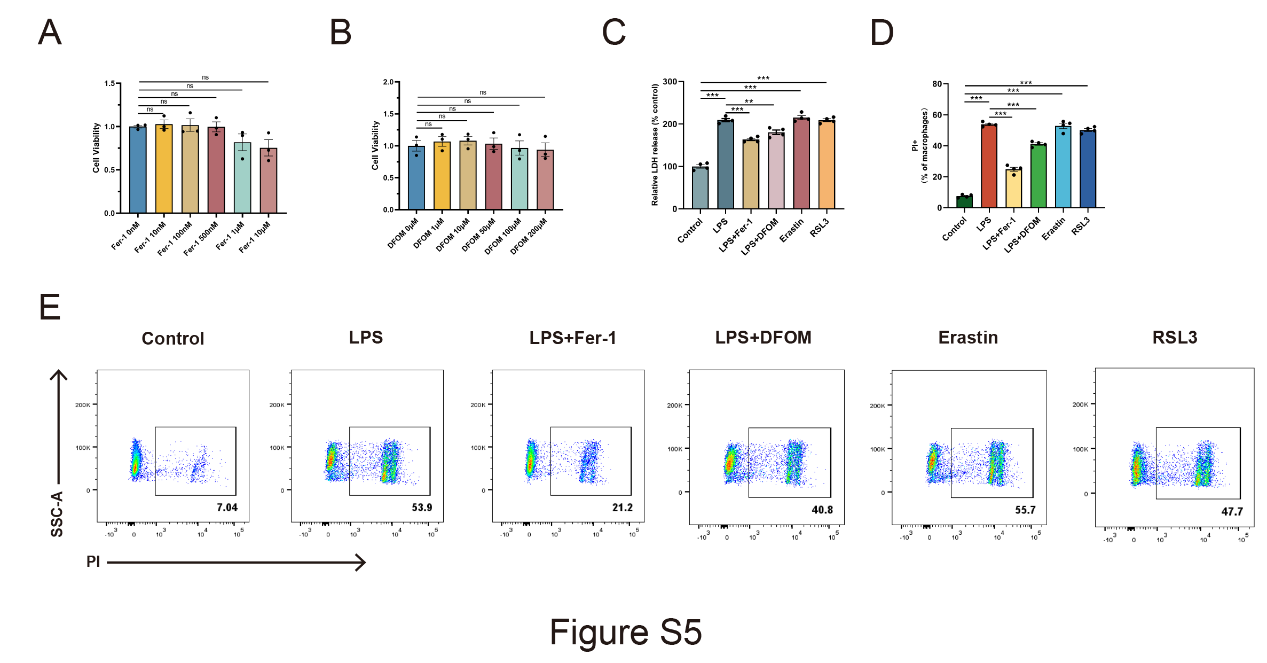


**Figure S5** Validation in BMDMs. (**A-B**) Cell viability of BMDMs treated with varying concentrations of ferrostatin-1 (Fer-1) or deferoxamine mesylate (DFOM) was assessed by CCK-8 assay. (**C-E**) BMDMs were pretreated for 30 min with Fer-1 (1 μM), DFOM (100 μM), erastin (5 μM), RSL3 (1 μM), followed by 24 h LPS stimulation (1 μg/mL). PI expression was detected by flow cytometry. The release of LDH in the supernatant of macrophages was detected. Data are mean±SEM from 4 (**C**, **D**) and 3 (**A**, **B**). *p < 0.05, **p < 0.01, ***p < 0.001.


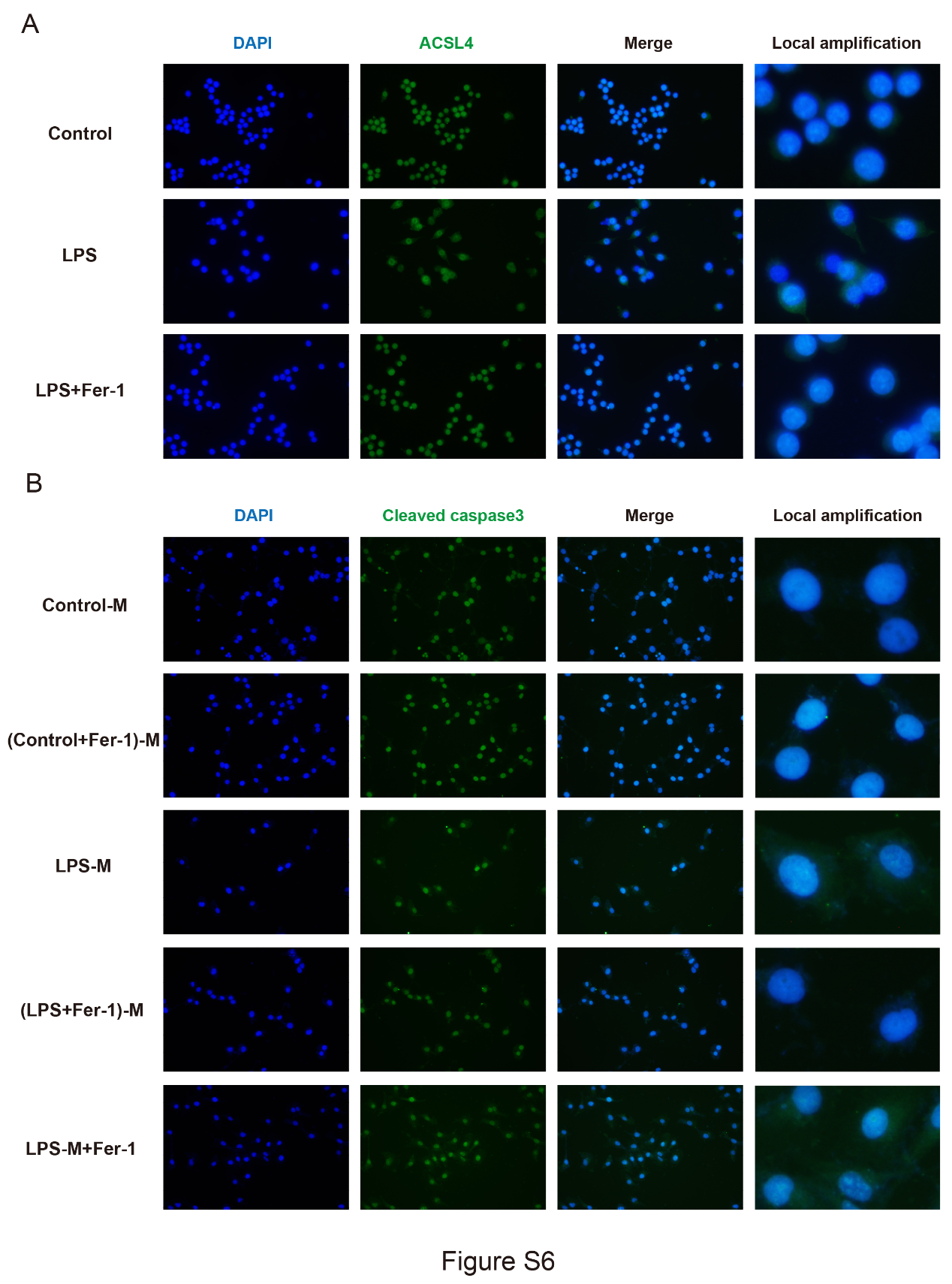


**Figure S6** Supplementary validation of macrophage ACSL4 and intestinal epithelial cell injury under different treatments. (**A**) Cell fluorescence was used to detect the expression of ACSL4 (green) in macrophages of control group, LPS group and LPS+Fer-1 group. (**B**) Cell fluorescence was used to detect the expression of cleaved caspase3 (green) in intestinal epithelial cells of control-M group, (control+Fer-1)-M group, LPS-M group, (LPS+Fer-1)-M group and LPS-M+Fer-1 group.


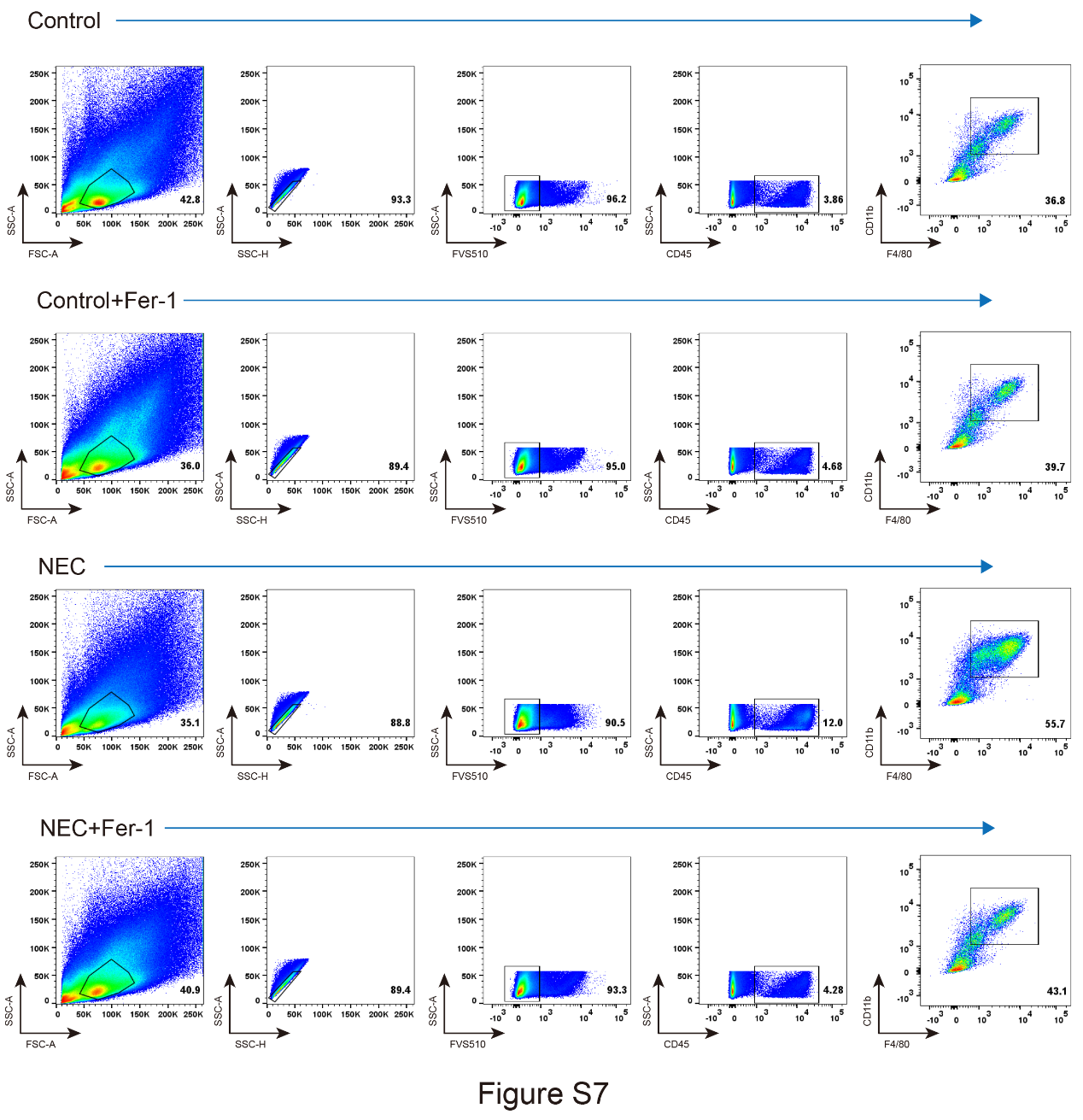


**Figure S7** Gating strategy schematic for mouse intestinal flow cytometry.
