# Supplementary figures and images for: Inhibition of ferroptosis in inflammatory macrophages alleviates intestinal injury in neonatal necrotizing enterocolitis
Source: Cell Death Discov. 2025 Aug 5;11:365. doi: 10.1038/s41420-025-02665-9 (PMC12325787; doi:10.1038/s41420-025-02665-9)

# Original blots with marker

Figure 5A

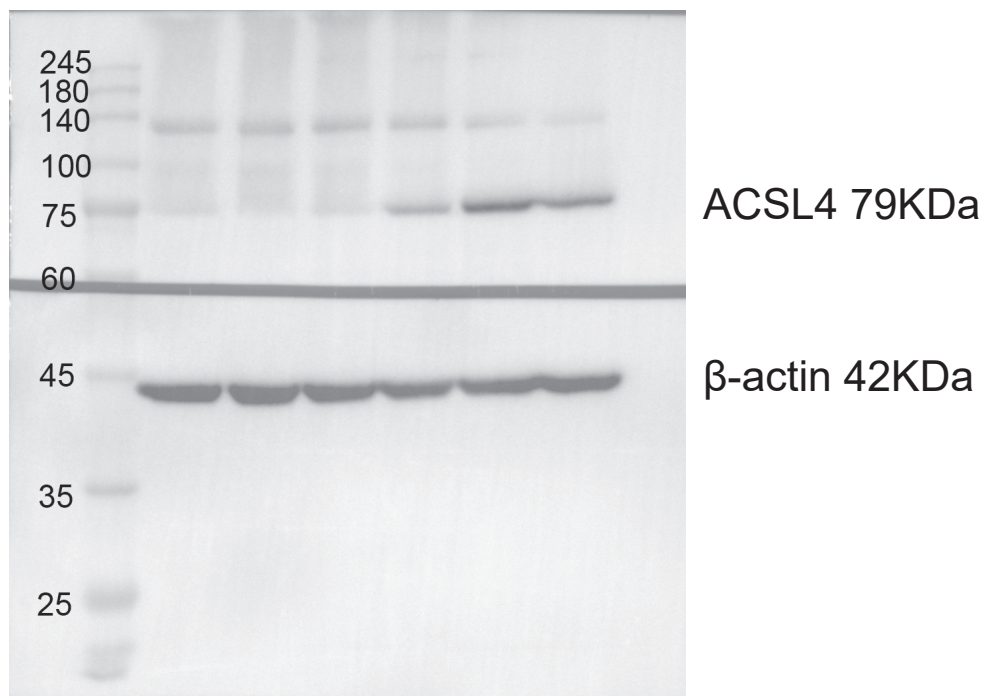

Figure 5G

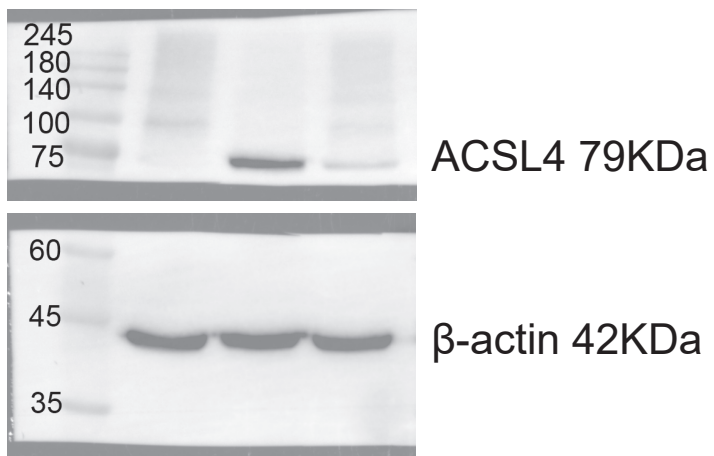

Figure 5I

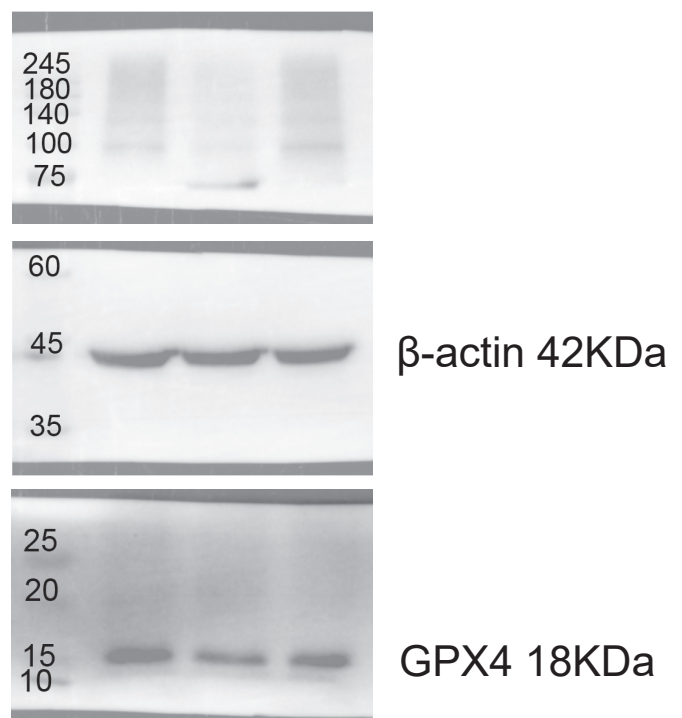

Figure 6D

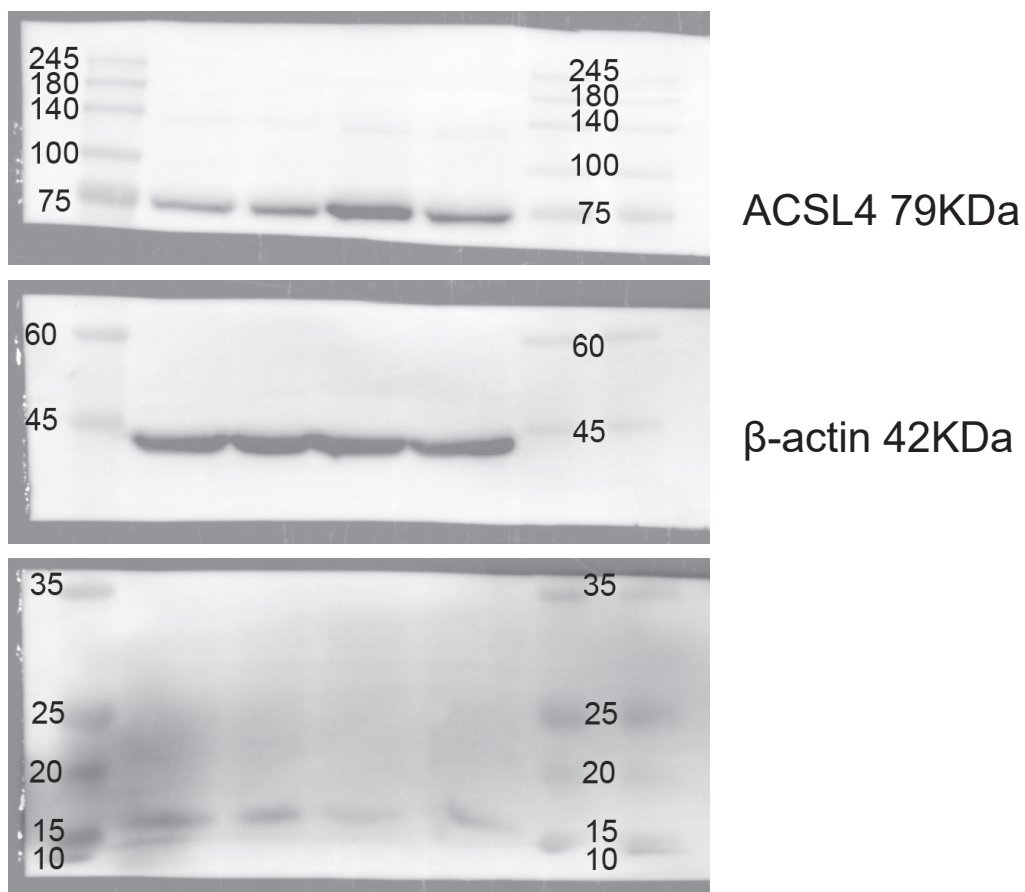

Figure 6F

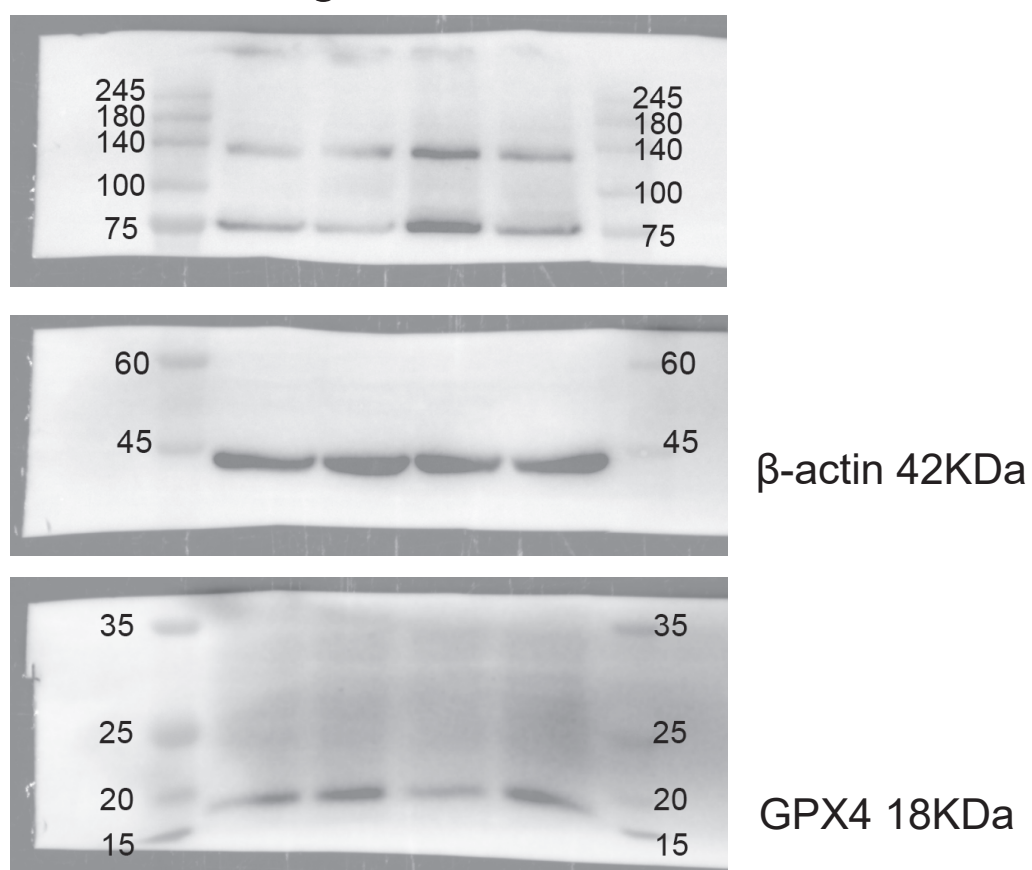

Supplement: Supplementary file 2 — Original blots [file 41420_2025_2665_MOESM2_ESM.pdf]
